# MAEA is an E3 ubiquitin ligase promoting autophagy and maintenance of haematopoietic stem cells

**DOI:** 10.1038/s41467-021-22749-1

**Published:** 2021-05-04

**Authors:** Qiaozhi Wei, Sandra Pinho, Shuxian Dong, Halley Pierce, Huihui Li, Fumio Nakahara, Jianing Xu, Chunliang Xu, Philip E. Boulais, Dachuan Zhang, Maria Maryanovich, Ana Maria Cuervo, Paul S. Frenette

**Affiliations:** 1 Ruth L. and David S. Gottesman Institute for Stem Cell and Regenerative Medicine Research, Bronx, NY USA; 2 Department of Cell Biology, Albert Einstein College of Medicine, Bronx, NY USA; 3 Department of Medicine, Albert Einstein College of Medicine, Bronx, NY USA; 4 Department of Development and Molecular Biology, Albert Einstein College of Medicine, Bronx, NY USA; 5 Human Oncology and Pathogenesis Program, Memorial Sloan Kettering Cancer Center, New York, NY USA; 6 Department of Anatomy and Structural Biology, Albert Einstein College of Medicine, Bronx, NY USA; 7 Institute for Aging Studies, Albert Einstein College of Medicine, Bronx, NY USA; 8 Present Address: Regeneron Pharmaceuticals, Inc., Tarrytown, NY USA; 9 Present Address: Department of Pharmacology, University of Illinois at Chicago, Chicago, IL USA; 10 Present Address: Department of Hematology and Oncology, Graduate School of Medicine, The University of Tokyo, Tokyo, Japan

**Keywords:** Macroautophagy, Cell signalling, Ubiquitin ligases, Haematopoietic stem cells

## Abstract

Haematopoietic stem cells (HSCs) tightly regulate their quiescence, proliferation, and differentiation to generate blood cells during the entire lifetime. The mechanisms by which these critical activities are balanced are still unclear. Here, we report that *Ma*crophage-*E*rythroblast *A*ttacher (MAEA, also known as EMP), a receptor thus far only identified in erythroblastic island, is a membrane-associated E3 ubiquitin ligase subunit essential for HSC maintenance and lymphoid potential. *Maea* is highly expressed in HSCs and its deletion in mice severely impairs HSC quiescence and leads to a lethal myeloproliferative syndrome. Mechanistically, we have found that the surface expression of several haematopoietic cytokine receptors (e.g. MPL, FLT3) is stabilised in the absence of *Maea*, thereby prolonging their intracellular signalling. This is associated with impaired autophagy flux in HSCs but not in mature haematopoietic cells. Administration of receptor kinase inhibitor or autophagy-inducing compounds rescues the functional defects of *Maea*-deficient HSCs. Our results suggest that MAEA provides E3 ubiquitin ligase activity, guarding HSC function by restricting cytokine receptor signalling via autophagy.

## Abstract (web summary)

Haematopoietic stem cells (HSCs) are metabolically quiescent, with balanced myeloid and lymphoid potential. Here the authors show that MAEA is required in HSCs for ubiquitination and downregulation of surface cytokine receptors via autophagy; MAEA loss leads to impaired HSC quiescence and a myeloproliferative disorder.

## Introduction

Under homoeostasis, haematopoietic stem cells (HSCs) reside in specialised bone marrow (BM) niches in a metabolically inactive quiescent state with balanced myeloid and lymphoid differentiation potential. Both intrinsic and extrinsic mechanisms have been proposed to regulate HSC maintenance and lineage potential^1–3^. Specialised (arterial and megakaryocytic) niches have been reported to promote quiescence of lineage-biased HSC subsets^4^, however progenitor differentiation and HSC maintenance also co-exist within overlapping niches^5–7^. How HSCs, often expressing the same cytokine receptors as their progeny (e.g. MPL, c-Kit), retain self-renewal and quiescence in overlapping niches while their progeny proliferate and differentiate, remains unclear. Autophagy (macroautophagy), a highly conserved process that recycles macromolecules and organelles via lysosomal degradation, has recently been shown to be critical for HSC quiescence and maintenance by controlling mitochondria homoeostasis, metabolic and oxidative stress^8,9^. But the molecular mechanism ensuring a higher level of autophagy in HSCs than their differentiated progeny^10^ is not clear and may provide insights on their unique quiescence-maintaining mechanisms.

Macrophage-Erythroblast Attacher (MAEA, also known as EMP) is an adhesion molecule only identified as essential for erythroblastic island formation^11^. Germline deletio*n* of *Maea* leads to severe anaemia and perinatal mortality^12^, but its function outside of erythroblastic island in postnatal animals remained unexplored. Recent phylogenetic and biochemical analyses have suggested that MAEA is a RING domain-containing subunit of a highly conserved E3 ubiquitin ligase complex with unknown functions in higher organisms^13,14^.

Here, we show that MAEA is essential for adult HSC maintenance. *Maea* deletion in mice lead to HSC loss and reduced lymphoid potential due to aberrant activation while sparing the mature haematopoietic cells. Mechanistically, MAEA is required for ubiquitination and downmodulation of surface cytokine receptor expression via autophagy, consistent with its role as an E3 ubiquitin ligase*. Maea* deletion prolongs receptor surface expression and impairs autophagy flux in HSCs, but not in their mature progeny, thus providing a mechanism guarding HSC quiescence and function by restricting cytokine receptor signalling and regulating autophagy.

## Results

### MAEA is required in HSCs for normal haematopoiesis

**Fig. 1:**
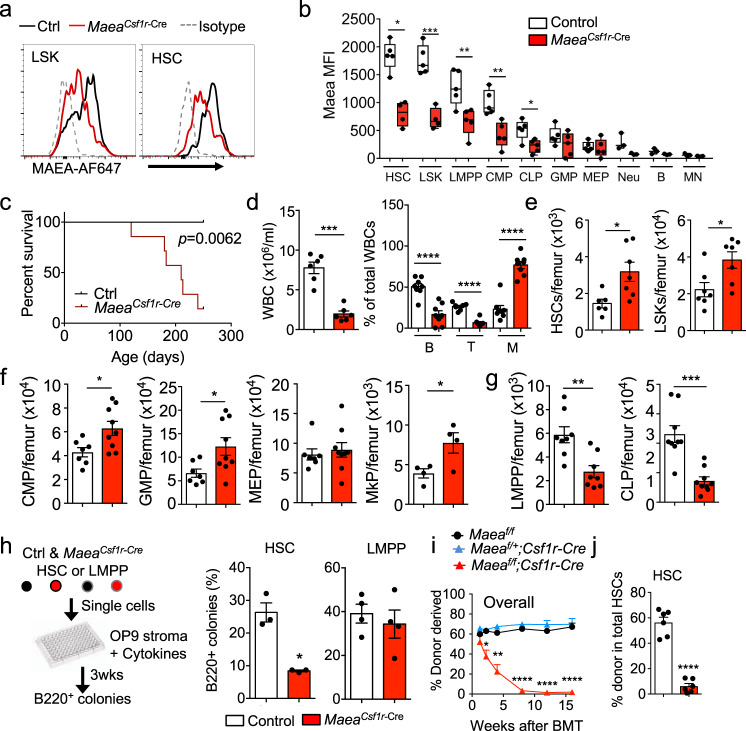
MAEA expression is enriched and required in haematopoietic stem cells (HSCs) for haematopoiesis. **a** Representative histograms showing MAEA expression on lineage- Sca-1 + c-kit+ progenitor cells (LSKs) and HSCs in control (Ctrl) and *Maea*^*Csf1r-Cre*^ bone marrow (BM). **b** FACS quantification of MAEA expression on control (*n* = 5) and *Maea*^*Csf1r-Cre*^ (*n* = 4) BM HSPCs (LMPP lymphoid-primed multipotent progenitors, CMP common myeloid progenitors, CLP common lymphoid progenitors, GMP granulocyte-macrophage progenitors, MEP megakaryocyte-erythrocyte progenitors), neutrophils (Neu), B cells (B) and monocytes (MN). Data are represented as boxes-and-whiskers with the whiskers span from minima to maxima of each dataset, the boxes extend from the 25th to 75th percentiles and the centre line indicates the mean. HSC *p* = 0.0202, LSK *p* = 0.0004, LMPP *p* = 0.0076, CMP *p* = 0.0016, CLP *p* = 0.0173. **c** The Kaplan–Meyer survival curve of control (*n* = 8) and *Maea*^*Csf1r-Cre*^ (*n* = 6) mice. *p* value was calculated by log-rank test. **d** White blood cell (WBC) counts in peripheral blood (*n* = 6 each group) and frequency of B, T and myeloid cells in total WBCs of young adult control and *Maea*^*Csf1r-Cre*^ mice (*n* = 8 each group). WBC *p* = 0.0001, B, T and M *p* < 0.0001. **e** Quantifications of HSC and LSK numbers in BM of control (*n* = 6) and *Maea*^*Csf1r-Cre*^ mice (*n* = 7). HSC *p* = 0.0165, LSK *p* = 0.024. **f** Quantification of myeloid progenitors in BM of control (*n* = 6) and *Maea*^*Csf1r-Cre*^ (*n* = 9) mice (MkP: *n* = 4). CMP *p* = 0.0146, GMP *p* = 0.0186, MkP *p* = 0.0499. **g** Quantification of lymphoid progenitors in BM of control (*n* = 6) and *Maea*^*Csf1r-Cre*^ (*n* = 9) mice^.^ LMPP *p* = 0.0027, CLP *p* = 0.0003. **h** Experimental scheme and results for evaluating lymphoid differentiation potential of HSC and LMPP at single cell level (HSC: *n* = 3, LMPP: *n* = 4 each group). *p* = 0.025. **i** Peripheral blood donor chimaerism at indicated time points after competitive BM transplantation (BMT) of equal number (1 × 10^6^) of CD45.1 wild-type (WT) competitor BM cells and CD45.2 donor BM cells from indicated genotypes into lethally irradiated CD45.1 WT recipients (fl/fl: *n* = 4, fl/+; Cre: *n* = 5; fl/fl; Cre: *n* = 6). 1.25 week *p* = 0.45, 2.25 week *p* = 0.027, 4 week *p* = 0.006, 8, 12 16 weeks *p* < 0.0001. **j** HSC donor chimaerism in the recipient BM from **i** at 16wks after transplant (*n* = 6 each group). *p* < 0.0001. All data are shown as mean ± sem and *p < 0.05, **p < 0.01, ***p < 0.001, ****p < 0.0001 by unpaired two-sided Student’s *t* test unless otherwise indicated.

Conditional *Maea* gene deletion recently revealed that MAEA expression on macrophages, but not erythroblasts, was required for postnatal EI formation^15^. Unexpectedly, we have found that MAEA was also expressed at high levels on HSCs and deleted at high efficiently (>80%) in haematopoietic stem and progenitor cells (HSPCs) using *Csf1r*-Cre (Supplementary Fig. 1a and Fig. 1a, b; hereafter referred to as *Maea*^*Csf1r-Cre*^). The recombination of *Csf1r*-Cre in HSCs was surprising as it is classically used to target mononuclear phagocytes^16^, but is consistent with the reported expression of *Csf1r* transcripts in HSPCs^17^. *Maea*^*Csf1r-Cre*^ mice are viable but die prematurely between 4 and 8 months of age with a myeloproliferative syndrome characterised by thrombocytosis, anaemia and increased infiltration of myeloid cells in fatal organs, such as liver and lung (Fig. 1c and Supplementary Fig. 1b, c). Examination of the BM at ~7 months of age revealed a near absence of B-lymphocytes with increased BM cellularity due to Gr-1^+^ cell expansion (Supplementary Fig. 1d, e). By contrast, young adult Maea^*Csf1r-Cre*^ mice did not exhibit anaemia or a myeloproliferative syndrome, but had a marked reduction (by ~75%) of circulating leucocytes due to severe lymphopenia (Fig. 1d). Analysis of their BM revealed a significant elevation of HSC numbers (CD150^+^ CD48^−^ lineage^−^ Sca-1^+^ c-kit^+^ LSKs) and myeloid progenitors in Maea^*Csf1r-Cre*^ BM compared to control animals, whereas the lymphoid progenitors were reduced (Fig. 1e–g and Supplementary Fig. 2a–d). However, we did not detect any alterations of myeloid-biased HSCs (CD150^hi^)^18,19^ in the *Maea*^*Csf1r-Cre*^ BM (Supplementary Fig. 2e). To obtain insight into whether *Maea* was required for lymphoid progenitor maintenance or HSC function, we sorted single lymphoid-primed multipotent progenitors (LMPPs) or HSCs from control and *Maea*^*Csf1r-Cre*^ BM onto OP9 stromal cells to examine their lymphoid differentiation potential. We found that *Maea*^*Csf1r-Cre*^ HSCs, but not the LMPPs, showed reductions in lymphoid differentiation (Fig. 1h). These results suggest that HSCs in *Maea*^*Csf1r-Cre*^ mice are skewed towards the myeloid lineage at the expense of the lymphoid potential.

To evaluate further *Maea*’s function in HSCs, we examined their ability to competitively repopulate the BM of lethally irradiated recipients. Surprisingly, despite a higher frequency of phenotypic HSCs, we observed a marked reduction in long-term repopulation of peripheral blood across all lineages from *Maea*^*Csf1r-Cre*^ donor cells compared to *Maea*^*fl/fl*^ and *Maea*^*fl/+*^; *Csf1r*-Cre^+^ control littermates (Fig. 1i and Supplementary Fig. 2f). Analysis of recipient BM at 16 weeks after transplantation confirmed the severe reduction in the HSC chimerism from *Maea*^*Csf1r-Cre*^ donors (Fig. 1j). However, we found no reduction of *Maea*^*Csf1r-Cre*^ cell homing to the BM (Supplementary Fig. 2g), suggesting that MAEA controls HSC repopulation activity. Since *Maea* deletion by *Csf1r*-Cre also results in BM macrophage defects^15^ and macrophages are a critical HSC niche component^5^, we analysed *Maea*^*CD169-Cre*^ mice in which *Maea* is selectively deleted in macrophages^15^. By contrast to *Maea*^*Csf1r-Cre*^ animals, we did not observe lymphopenia in peripheral blood or BM of these mice (Supplementary Fig. 3a–c). In addition, there was no alteration in the numbers of HSC and progenitors (Supplementary Fig. 3d–f). To evaluate the role of MAEA in the haematopoietic vs the stromal compartment, we carried out reciprocal transplantation experiments in which wild-type (WT) BM cells were transplanted into lethally irradiated *Maea*^*Csf1r-Cre*^ mice and vice versa (Supplementary Fig. 3g–l). We only detected lymphopenia in WT recipients transplanted with *Maea*^*Csf1r-Cre*^ BM, while WT donor BM completely normalised the peripheral blood indexes of *Maea*^*Csf1r-Cre*^ mice. Taken together, these results indicate that the defects of *Maea*^*Csf1r-Cre*^ mice have an HSC autonomous origin and are not related to gene deletion in macrophages.

### MAEA guards HSC quiescence in a mTOR-dependent manner

**Fig. 2:**
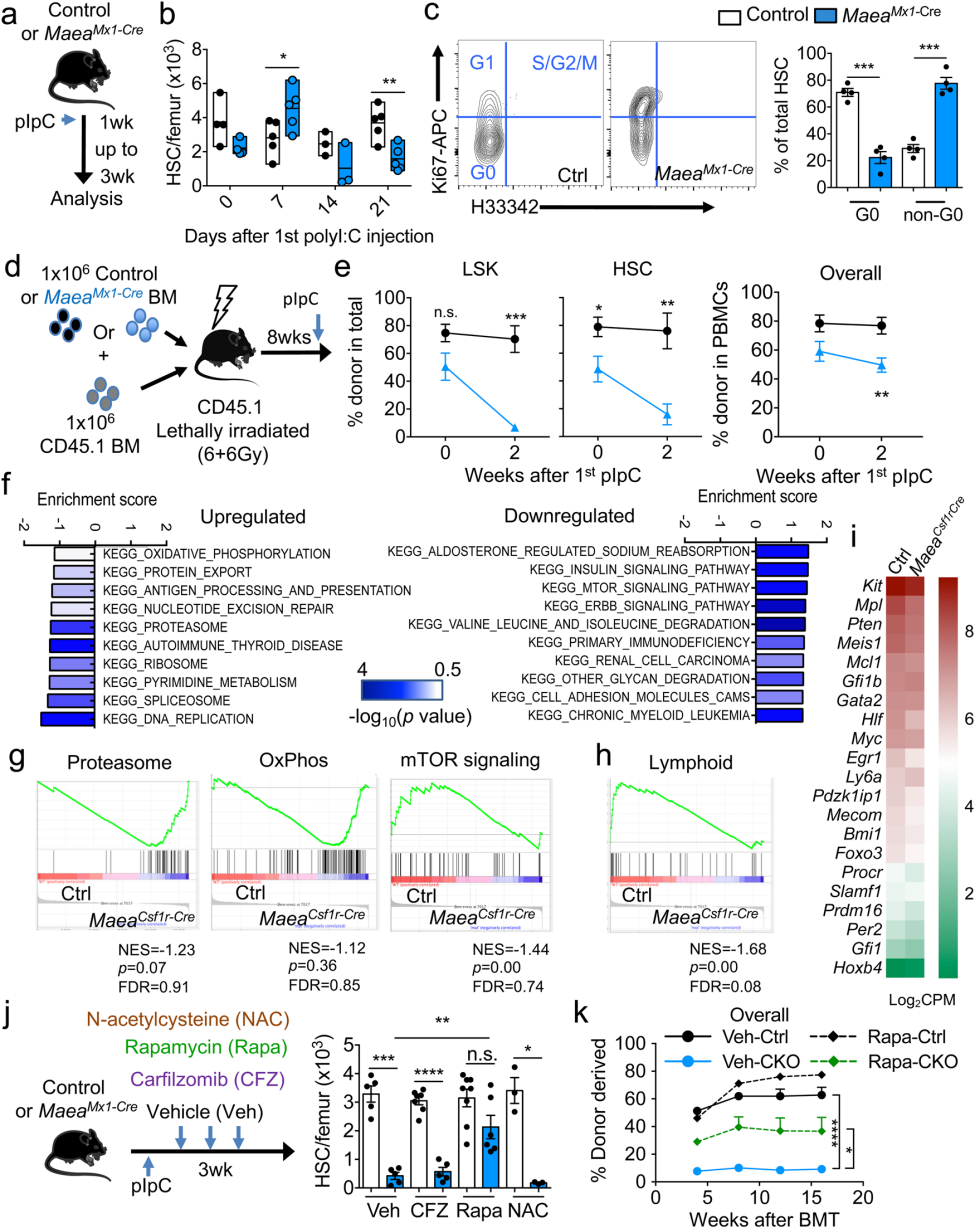
*Maea* deletion impairs haematopoietic stem cells (HSCs) quiescence and function in a mTOR-dependent manner. **a** Experimental scheme for deleting *Maea* in adult mice using *Mx1*-Cre. (The mouse symbol in these figures were modified and recreated from Servier Medical Art (https://smart.servier.com/), an open source of medical images). **b** Quantification of bone marrow (BM) HSC numbers at indicated time points after poly I:C injection (day 0: *n* = 4; days 7 and 21: *n* = 5; day 14: *n* = 3 each group). Data are represented as floating boxes with boundaries indicate minima to maxima of each dataset and middle line indicates the mean. Day 0 *p* = 0.069, day 7 *p* = 0.0457, day 14 *p* = 0.168, day 21 *p* = 0.005. **c** Representative FACS plots and cell cycle profiles of control (Ctrl) and *Maea*^*Mx1-Cre*^ (CKO) HSCs at 21 days after 1st poly I:C injection (*n* = 4). *p* = 0.0002. **d** Experimental scheme for deleting *Maea* in 1:1 wild-type (WT) and *Maea*^*Mx1-Cre*^ BM chimeras after stable (8 weeks) reconstitution. **e** Donor chimaerism in BM LSK and HSCs and peripheral blood total leucocytes from control and *Maea*^*Mx1-Cre*^ mixed chimeric mice at indicated time points after poly I:C injection (*n* = 10 over two independent experiments). **f** RNA-seq and Gene Set Enrichment Analysis (GSEA) of HSCs from BM of control and *Maea*^*Csf1r-Cre*^ young adults at 7–12 weeks of age. Three replicates with 2000 HSCs pooled from two mice each replicate were processed and analysed for each group. Top KEGG (Kyoto Encyclopaedia of Genes and Genomes) pathways that are up-regulated and down-regulated in *Maea*^*Csf1r-Cre*^ HSCs are shown. **g** Examples of GSEA enrichment plots showing enrichment of Proteasome, Oxidative phosphorylation and mTOR signalling pathways in *Maea*^*Csf1r-Cre*^ HSCs. **h** GSEA enrichment plot showing significant downregulation of lymphoid potential related gene set in *Maea*^*Csf1r-Cre*^ HSCs. **i** Heat map showing mean expression of HSC related genes in control and *Maea*^*Csf1r-Cre*^ HSCs. **j** Experimental scheme and quantification of LSKs and HSCs in control and *Maea*^*Mx1-Cre*^ mice treated with vehicle (Veh), carfilzomib (CFZ), rapamycin (Rapa) or N-acetylcysteine (NAC) for 3 weeks after poly I:C induction (Veh: *n* = 5, CFZ: *n* = 6, Rapa: *n* = 7, NAC: *n* = 3 over two independent experiments). **k** Donor chimaerism in peripheral blood of CD45.1 lethally irradiated wild-type recipients at indicated time points after competitive BM transplantation (BMT) of equal number of CD45.1 WT competitor BM cells and CD45.2 donor BM cells from indicated groups (*n* = 5 each group). All data are mean ± sem unless otherwise indicated. *p < 0.05, **p < 0.01, ***p < 0.001, ****p < 0.0001 by unpaired two-sided *t*-test with 95% confidence level. ns not significant.

We next evaluated HSC function after *Maea* deletion using the conditional *Mx1*-Cre line (*Maea*^*Mx1-Cre*^) and poly I:C administration (Fig. 2a and Supplementary Fig. 4a). During the time course of 3 weeks after the first poly I:C injection, we found that *Maea*^*Mx1-Cre*^ HSCs initially expanded at day 7 followed by a significant reduction compared to control *Maea*^*f/f*^ mice (Fig. 2b), while the total BM cellularity was not altered (Supplementary Fig. 4b). Cell cycle analysis revealed a dramatic loss of quiescence of *Maea*^*Mx1-Cre*^ HSCs (Fig. 2c), but not of other cell populations after poly I:C induction (Supplementary Fig. 4c). This was associated with a preferential loss of CD150^lo^ HSCs and a significant reduction of lymphoid progenitor cells but an increase in neutrophil numbers (Supplementary Fig. 4d, e). Although no mortality was observed in these *Maea*^*Mx1-Cre*^ mice ~4 months after poly I:C, we detected mild anaemia, myeloid expansion and significantly decreased BM HSC numbers resembling the *Maea*^*Csf1r-Cre*^ mice (Supplementary Fig. 4f). In addition, *Maea*^*Csf1r-Cre*^ HSCs were also more actively cycling in young mice while their numbers were depleted in older mice (Supplementary Fig. 4g, h). These results suggest *Maea* deletion depletes HSCs by aberrant activation, followed by their exhaustion.

To evaluate further the HSC-intrinsic requirement of MAEA, we generated chimeric mice by transplantation of an equal mixture of WT (CD45.1) and *Maea*^*Mx1-Cre*^ (CD45.2) BM cells into lethally irradiated WT (CD45.1) recipients and induced *Maea* deletion after stable reconstitution (Fig. 2d). Analysis of the BM and peripheral blood donor chimerism showed a drastic reduction of *Maea*^*Mx1-Cre*^ HSCs and LSKs in the chimeric BM in the presence of WT competitor cells at 2 weeks and a lower peripheral contribution over 8 weeks after poly I:C induction (Fig. 2e and Supplementary Fig. 4i). Thus, these results clearly support an intrinsic role of *Maea* in HSC maintenance.

We then interrogated the transcriptome of sorted *Maea*^*Csf1r-Cre*^ and littermate control HSCs to gain mechanistic insight on how *Maea* regulated HSC function. Gene Set Enrichment Analysis (GSEA) revealed a striking up-regulation of gene sets involved in cell activation or proliferation in *Maea*^*Csf1r-Cre*^ HSCs, such as DNA replication, protein synthesis/processing and oxidative phosphorylation, but downregulation of several major cell growth-related pathways, including insulin and mTOR signalling (Fig. 2f, g and Supplementary Fig. 4j). Consistent with the defective lymphoid and engraftment potential, the expression of regulators of HSC lymphoid potential^20^ and maintenance was reduced (Fig. 2h, i). The transcriptional downregulation of cell growth-related pathways was counterintuitive in activated HSCs. Since the activity of these pathways could be critically regulated at post-translational levels, we assessed their downstream signalling molecules by intracellular phospho-flow cytometry. We detected a significant downregulation of total ribosomal protein S6, a downstream target of mTOR, in *Maea*^*Csf1r-Cre*^ HSCs compared to control, and a milder reduction of the phosphorylated S6 (pS6 Ser235/236). This resulted in a significant increase in the pS6/S6 ratio (Supplementary Fig. 4k), suggesting hyper-activity of mTORC1 signalling. We did not observe any significant changes in pAkt Ser473 or pErk1/2 Thr202/Tyr204 basal levels (Supplementary Fig. 4l).

Based on these analyses, we assessed the functional significance of these enriched pathways by treating poly I:C-induced *Maea*^*Mx1-Cre*^ mice with either a proteasome inhibitor (Carfilzomib (CFZ)), an inhibitor of oxidative stress (N-acetylcysteine (NAC)), or a mTOR antagonist (rapamycin). Remarkably, while none of the inhibitors significantly altered BM cellularity, rapamycin, but not CFZ or NAC, rescued the HSC numbers after *Maea* deletion (Fig. 2j and Supplementary Fig. 5a, b). Competitive transplantation experiments from treated and control mice also confirmed the rescue of functional HSC activity in rapamycin-treated *Maea*^*Mx1-Cre*^ mice (Fig. 2k and Supplementary Fig. 5c). Rapamycin, however, did not rescue macrophage numbers in BM due to *Maea* deletion (Supplementary Fig. 5d)^15^, suggesting cell-type-specific functions of MAEA. Our results thus suggest that MAEA suppresses mTOR activity in HSCs.

### MAEA regulates cytokine receptor stability in HSCs

**Fig. 3:**
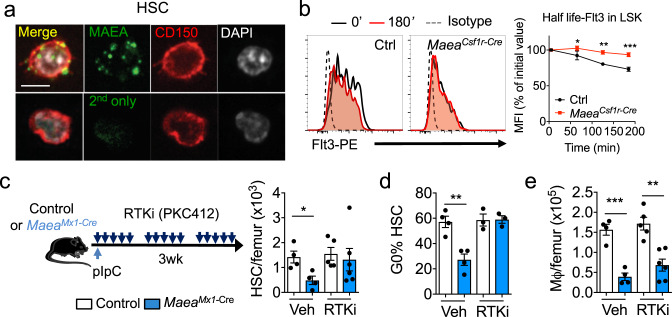
MAEA regulates cytokine receptor ubiquitination and stability in hematopoietic stem cells (HSCs). **a** Representative immunofluorescence images showing MAEA and CD150 expression in HSCs using cells from two independent experiments of four mice. Scale bar = 10 μm. **b** Representative histograms and FACS evaluation of Flt3 half-life in control and *Maea*^*Csf1r-Cre*^ LSK cells incubated in the presence of 50 μM cycloheximide (*n* = 4 animals). *p < 0.05, **p < 0.01, ***p < 0.001 by two-way ANOVA multiple comparisons. exact *p* value from left to right are 0.0325, 0.0016, 0.0003. **c** Experimental scheme and quantification of HSCs in control (*n* = 9) and *Maea*^*Mx1-Cre*^ (*n* = 10) mice treated with vehicle and RTK inhibitor (RTKi) PKC412 for 3 weeks after poly I:C induction. *p* = 0.0212. **d** Quantification of the frequencies of G0 phase HSCs in control and *Maea*^*Mx1-Cre*^ (*n* = 7 each) mice treated with vehicle and RTKi PKC412 at 3 weeks after poly I:C induction. *p* = 0.0033. **e** Quantification of BM macrophages in control (*n* = 9) and *Maea*^*Mx1-Cre*^ (*n* = 10) mice treated with vehicle and RTKi PKC412 at 3 weeks after poly I:C induction. *p* value from left to right are 0.0007, 0.0012. All data are shown as mean ± sem. ns not significant. *p < 0.05, **p < 0.01, ***p < 0.001, ****p < 0.0001 by unpaired two-sided *t-*test with 95% confidence level unless otherwise indicated.

We next sought to identify how MAEA could interfere with mTOR/intracellular signalling. Although previous studies have suggested that MAEA might be expressed in the nucleus and/or associated with the actin filaments^12,21,22^, immunofluorescence analysis of permeabilized HSCs detected MAEA expression only at the cell surface in localised foci (Fig. 3a), raising the possibility that it might be involved at surface signalling centres. Recent phylogenetic and biochemical analyses have suggested that MAEA is a RING domain-containing subunit of a highly conserved E3 ubiquitin ligase complex with unknown functions in higher organisms^13,14^. We thus examined the ubiquitination landscape in *Maea*-deficient and sufficient HSPCs using a ubiquitin antibody array. We found that ubiquitinated targets comprising several cell surface receptors were significantly reduced in *Maea*^*Csf1r-Cre*^ lineage-negative BM cells compared to control while three targets (Caspase-8, F-box protein 15 and p21Cdkn1a) were significantly increased (Supplementary Fig. 6a). Ubiquitin modifications of protein substrates may lead to proteasome or lysosome-dependent substrate degradation or may modulate substrate interactome and subcellular localisation^23^. Importantly, ubiquitination regulates sorting, trafficking and removal of membrane proteins via endocytosis and plays a critical role in fine-tuning their physiological functions^24,25^. To further investigate the functional impact of *Maea* deletion on receptor ubiquitination, we focused on the half-life and downstream signalling of MPL, c-kit and FLT3, major receptors regulating haematopoiesis. Aberrant activation of these receptors has been associated with myeloproliferative disease involving the HSPCs^26–29^. We treated BM cells ex vivo with the translation inhibitor cycloheximide and evaluated the turnover of surface receptors in *Maea*^*Csf1r-Cre*^ and control HSPCs. We found that the half-lives of FLT3 and MPL, but not c-kit, were significantly prolonged in *Maea*^*Csf1r-Cre*^ HSPCs (Fig. 3b and Supplementary Fig. 6b). We also detected elevated pSTAT5a levels in *Maea*^*Csf1r-Cre*^ LSKs (Supplementary Fig. 6c), and a persistence of pAKT, but not pERK, signal in HSPCs after cytokine stimulation, despite comparable constitutive levels (Supplementary Fig. 6d). These results suggest that MAEA may negatively modulate receptor tyrosine kinase signalling in HSPCs by promoting receptor ubiquitination and degradation. Indeed, when ectopically expressed, MAEA was able to promote poly-ubiquitination of FLT3 (Supplementary Fig. 6e) indicating that FLT3 is a substrate for its ligase activity. Additionally, a receptor tyrosine kinase inhibitor (RTKi, PKC412/Midostaurin) that has been clinically proven to inhibit FLT3 signalling^30,31^, lowered the pSTAT5a levels (Supplementary Fig. 6c), rescued HSC depletion and loss of quiescence but not macrophage reduction upon *Maea* deletion (Fig. 3c–e). These results indicate that MAEA-mediated receptor ubiquitination and signal restriction plays a critical role in HSC quiescence and maintenance.

### MAEA regulates cytokine receptor stability via autophagy

**Fig. 4:**
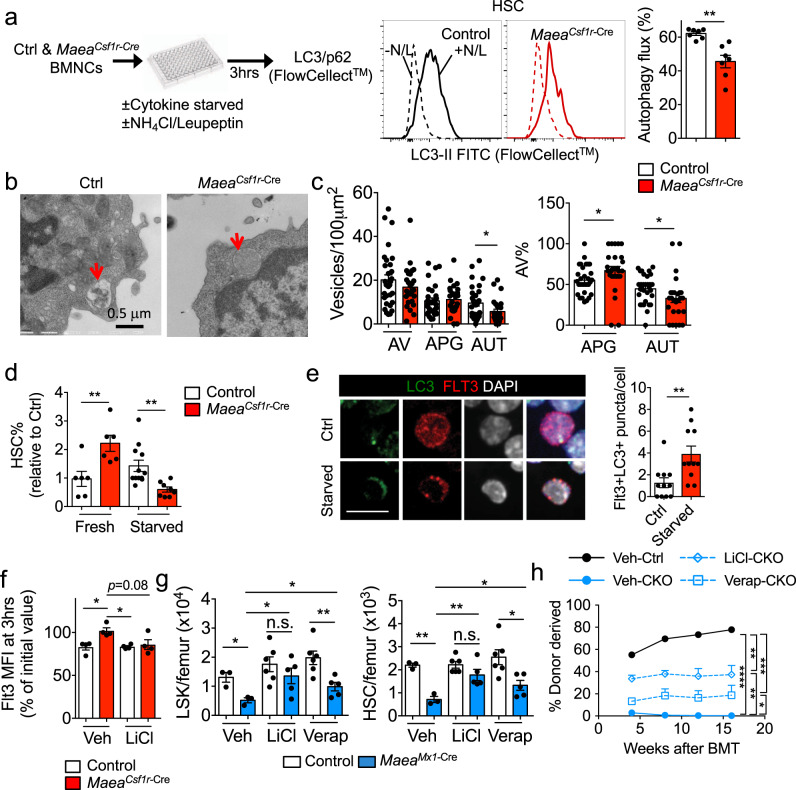
MAEA regulates cytokine receptor stability via autophagy in hematopoietic stem cells (HSCs). **a** Experimental scheme and evaluation of autophagy flux in control (Ctrl) and *Maea*^*Csf1r-Cre*^ HSCs (*n* = 7). % autophagy flux is calculated as 100x(1 − (−L/N))/(+L/N). N: NH_4_Cl. L: leupeptin. *p* = 0.0036. **b** Representative electron microscopy micrographs of control and *Maea*^*Csf1r-Cre*^ HSCs for ultrastructural analysis of the autophagic compartments (red arrows point to an autolysosome in control and an autophagosome in *Maea*^*Csf1r-Cre*^). **c** Morphometric analysis of control and *Maea*^*Csf1r-Cre*^ HSCs: quantification of the numbers of autophagic vacuoles (AV) and their break down into number (left) and percentage (right) of autophagosomes (APG) and autolysosomes (AUT), per cell area. *n* = 27 control and 23 *Maea*^*Csf1r-Cre*^ cells analysed. Analysis were applied to cells blindly from two independent experiments. Unblinding was done during data plotting. *p* value from left to right are *p* = 0.035, 0.0245, 0.0306. **d** Frequencies of HSCs in control and *Maea*^*Csf1r-Cre*^ bone marrow (BM) cells before and after 3 h of starvation in culture (*n* = 6 each). *p* value from left to right are 0.0093, 0.0016. **e** Representative immunofluorescence images and quantification showing subcellular colocalization of FLT3 and LC3 in freshly isolated (Ctrl) and starved (cultured ex vivo in StemSpan with no cytokines but in the presence of lysosome inhibitors N/L for 3 h to induce autophagy) HSCs (*n* = 11 cells analysed). *p* = 0.0071. Scale bar = 10 μm. **f** Flt3 half-life in control and *Maea*^*Csf1r-Cre*^ lineage- Sca-1+ c-kit+ progenitor cells (LSKs) cells incubated in the presence of 50 μM cycloheximide and 10 mM lithium chloride (LiCl) (*n* = 4 animals). *p* value from left to right are 0.034, 0.0105, 0.08. **g** Quantification of LSKs and HSCs in control and *Maea*^*Mx1-Cre*^ BM treated with vehicle (Veh), LiCl or verapamil (Verap) 3 weeks after poly I:C induction (Veh: *n* = 3, LiCl: *n* = 6, Verap: *n* = 6). **h** Peripheral blood donor chimaerism in CD45.1 lethally irradiated wild-type (WT) recipients at indicated time points after competitive BM transplantation (BMT) of equal number of CD45.1 WT competitor BM cells and CD45.2 donor BM cells from indicated groups (*n* = 5 each group). Data are shown as mean ± sem. ns not significant. *p < 0.05, **p < 0.01, ***p < 0.001, ****p < 0.0001 by unpaired two-sided *t*-test with 95% confidence level unless otherwise indicated.

Although the mTOR inhibitor rapamycin rescued the HSC depletion and loss of quiescence induced by *Maea* deletion, MAEA did not directly regulate mTOR signalling (Supplementary Fig. 6d, f, g). We thus asked if rapamycin could rescue *Maea*-deficient HSCs via the promotion of lysosome-dependent degradation pathways (e.g. autophagy). Macroautophagy, a highly conserved mechanism to recycle macromolecules and organelles via lysosomal degradation, was suggested to be critical for HSC quiescence and maintenance although the detailed molecular mechanism have remained unclear^9,10,32,33^. mTOR signalling and activated AKT are reported to inhibit autophagy, and rapamycin is a potent inducer of autophagy^34,35^. Our transcriptomic profiling indicated no significant change in the expression of the core autophagy machinery or pro-autophagy genes in *Maea*-deficient and sufficient HSCs (Supplementary Fig. 7a, b). When cultured under starvation conditions or in the presence of rapamycin, WT HSCs exhibited high levels of autophagy flux of endogenous LC3-II (distinguishable from LC3-I through a permeabilization step) compared to more differentiated cells (Fig. 4a and Supplementary Fig. 7c), which is in line with previous studies^10,36^. However, the autophagy flux in *Maea*^*Csf1r-Cre*^ HSCs was significantly reduced (Fig. 4a). Lineage-negative cells (enriched in HSPCs), but not lineage-positive cells (enriched in mature haematopoietic cells), from *Maea*^*Csf1r-Cre*^ BM also presented significant reductions in LC3-II flux (Supplementary Fig. 7c). In addition, morphometric analyses using electron microscopy imaging confirmed that the reduced flux was largely due to a maturation defect in the autophagic compartment of *Maea*^*Csf1r-Cre*^ HSCs^37,38^, as we have found a significant reduction in the percentage of autolysosomes (AUT) relative to autophagosomes (APG; Fig. 4b, c and Supplementary Fig. 7d) in these cells. Interestingly, *Maea*^*Csf1r-Cre*^ HSCs also had a defect in autophagy induction (APG biogenesis) that can explain why APG did not accumulate in these cells despite the observed maturation defect (Fig. 4c). Reduced autophagy flux likely accounts for the lower survival rate of *Maea*-deficient HSCs upon starvation (Fig. 4d)^39^.

To ascertain whether the reduced autophagy flux was mechanistically linked with impaired receptor degradation in *Maea*^*Csf1r-Cre*^ HSPCs, we firstly examined the subcellular localisation of FLT3 and MPL and found both receptors colocalized with starvation-induced LC3 puncta (Fig. 4e and Supplementary Fig. 8a), suggesting that these receptors are subjected to degradation in the APG. Furthermore, rapamycin and mTOR-independent autophagy inducers (lithium^40^ or verapamil^41^), which promoted autophagy flux in *Maea*-deficient cells (Supplementary Fig. 8b, c), were able to normalise the prolonged FLT3 half-life in *Maea*-deficient HSPCs (Fig. 4f). Finally, we administered these mTOR-independent autophagy inducers to poly I:C-induced *Maea*^*Mx1-Cre*^ and control mice in vivo and found that both autophagy inducers could rescue HSC numbers and their repopulation activity (Fig. 4g, h and Supplementary Fig. 8d, e). These results thus identify MAEA as a critical regulator of HSC maintenance by enhancing ubiquitination of cytokine receptors and promoting autophagy.

## Discussion

Ubiquitination of cellular proteins fine-tunes their expression levels, cellular localisation, and interaction dynamics, thereby influencing many cellular processes^42^. Ligand-bound receptors are subject to endocytosis followed by recycling to the cell surface or autophago-lysosome degradation which is determined by their ubiquitination status^24,25^. Components of the ubiquitin proteasome system, mostly E3 ligases, have been suggested to regulate HSC fate by targeting signal transducers and transcription regulators^43^. Additionally, ubiquitination is emerging as an important autophagy regulator in other cell systems mostly by influencing the stability and interaction of the autophagy core machinery and selective cargo recognition^44,45^. We show here that ubiquitination and autophagy are functionally linked in the haematopoietic system.

We have identified MAEA as an E3 ubiquitin ligase subunit critical for HSC quiescence and lymphoid potential. Our data indicate that MAEA-mediated ubiquitination targets surface cytokine receptor tyrosine kinases for degradation in the AUT, hence fine-tunes their downstream signalling strength and duration. Interestingly, Maea was also required for normal APG maturation in the HSPCs potentially by promoting the cargo input into the APG and indirect suppression of mTOR signalling which could potently inhibits autophagy^34^, but not so in the mature progeny. These results clearly demonstrate a unique mechanism mediated by MAEA that links the ubiquitin system, autophagy and HSC quiescence/maintenance. Our results have implications in how the behaviour of HSCs can be distinguished from their progeny in overlapping BM niches where cytokines may evoke the maintenance of HSCs while at the same time promoting the proliferation and differentiation of progenitors^5–7^.

## Methods

### Animals

*Maea*^*fl/fl*^ mice were previously described and routinely genotyped and tested for knockout efficiency using primers listed in Supplementary Table 1^15^. *Csf1r*-iCre mice^16^ were a gift from Dr. Jeffrey W. Pollard (University of Edinburgh), and backcrossed onto C57BL/6 background. C57BL/6 (CD45.2) and Bl6-Ly5.1 (CD45.1) mice were purchased from Charles River Laboratories (Frederick Cancer Research Center, Frederick, MD)/NCI or the Jackson Laboratories (B6.SJL-*Ptprc*^*a*^ *Pepc*^*b*^/BoyJ). *Mx1*-Cre ﻿(B6.Cg-Tg(Mx1-cre)1Cgn/J) mice were obtained from The Jackson Laboratory and *CD169*-Cre mice have been previously described^15,46^. All animals were housed in specific pathogen-free barrier facility that is under a 12 h:12 h light/dark cycle and is temperature and humidity regulated. All experimental procedures were compiled with ethical regulations of animal testing and research and approved by the Animal Care and Use Committee of Albert Einstein College of Medicine. All experiments were performed on mice of both genders with littermate controls from the same colony between 6 and 12 weeks of age unless otherwise indicated.

### Antibodies and flow cytometry

BM cells were isolated by flushing long bones with 1 ml of ice-cold PEB (phosphate-buffered saline [PBS]/2 mM EDTA/0.5% bovine serum albumin) buffer through a 1-ml syringe (BD) with a 21G needle (BD) into fluorescence-activated cell sorting (FACS) tubes. Purified goat anti-MAEA polyclonal antibody (I-20) was purchased from Santa Cruz and used at 1:100 concentration. Conjugated donkey anti-goat IgG secondary antibodies were from Thermo Fisher and used at 1:800 concentration. An anti-MAEA monoclonal antibody (92.25) was generated by our laboratory and recently described^15^. Fluorochrome-conjugated or biotinylated antibodies against mouse F4/80-PE (clone BM8; Catalogue# 123110), CD115-PE/C7 (AFS98; 25-1152-82), B220-APC-eFluor780 (RA3-6B2; 47-0452-82), anti-CD3e-PerCP-Cy5.5 (145-2C11; 45-0031-82), Gr-1(Ly6C/G)-FITC (RB6-8C5; 11-5931-85), CD11b-PE (M1/17; 12-0112-83), CD45.1-PE/Cy (A20; 25-0453-82), CD45.2-FITC (104; 109806), F4/80 (clone BM8), CD45 (clone 30-F11), c-kit/CD117-PE/Cy7 (2B8; 105814), Sca-1-FITC (clone D7, 11-5981-85), CD150-PE (clone TC15-12F12.2, 115904), CD48-PerCP-eFluor710 (HM48-1; 46-0481-85), CD16/32- APC/CY7 (clone 93, 101328), CD34-eFlour660 (clone RAM34, 50-0341-82), CD41-FTIC (MWReg30, 11-0411-82), Flt3 (A2F10), CD127-PerCP/Cy5.5 (A7R34, 45-1271-82), Ki-67 PE-Cy7 (clone SolA15, 25-5698-80) were from BioLegend or eBiosciences. Biotin-c-Mpl/TPOR (AMM2, 10403) was from Immuno-Biological Laboratories. Biotinylated lineage cocktail (559971) and FITC anti-active Caspase-3 apoptosis kit (550480) were from BD Biosciences. FlowCellect™ Autophagy LC3 Antibody-based Assay Kit was from EMD Millipore (FCCH100171). DAPI-negative singlets were analysed for all live samples. For intracellular antigen detection, cells were fixed and permeabilized using BD Cytofix/Cytoperm Fixation and Permeabilization kit (#554714) after surface receptor staining and followed by antibody staining for intracellular markers in the Cytoperm/Cytowash buffer. For cell cycle analysis, DNA content was labelled by Hoechst 33342 (Sigma). For phospho-flow, cells were kept on ice upon isolation and immediately fixed in 1.5% PFA for 10 min. Cells were then washed and stained for cell surface markers. After surface staining, the cells were permeabilized with ice-cold acetone for 10 min on ice, washed and stained with the following phospho-specific antibodies: pS6 (S235/236; D57.2.2E, 4851S), S6 (54D2, 55594), p4EBP1 (T37/46; 236B4), p44/42 MAPK (T202/Y204; E10, 4375S), pAKT (S473; D9E, 5315S), all from Cell Signalling, were used at 1 μg/ml. All other antibodies were used at 1:100 dilutions unless otherwise indicated. Stained sample suspensions were acquired on a LSR II (BD) using FACSDIVA software (V4.1, BD Biosciences) and results were analysed and visualised by FlowJo 10.4.0 (LLC). For sorting, samples were processed under sterile conditions and sorted on a BD FACSAria.

### Complete blood count

Mice were bled ~25 μl into an Eppendorf tube containing 2 μl of 0.5 M EDTA (Life Technologies) using heparinised micro-haematocrit capillary tubes (Fisherbrand) under isoflurane anaesthesia. Blood was diluted 1:20 in PBS and analysed on an Advia counter (Siemens).

### Single cell lymphoid differentiation

The B-cell differentiation of single cells was done as previously described^47^. Briefly, the day before sorting, 96-well plates were pre-coated with 1.5 × 10^3^/well OP9 stromal feeder cells (ATCC CRL-2749) in OP9 base media (alpha MEM + 10% FCS). On the next day, single HSCs or LMPPs were directly sorted into the wells containing lymphoid differentiation media (OP9 base media + 25 ng/ml SCF, 50 ng/ml Flt3l, 100 ng/ml IL-7) on a FACSAria. The co-culture was maintained for 3 weeks at 37 °C in 5% CO_2_ with half-media change every 3–4 days. At the end of the co-culture, the wells were carefully washed and suspended in FACS buffer and stained for CD45, B220 and CD19 before analysis on a CantoII high throughput analyser.

### Bone marrow transplantation

All recipient mice were lethally irradiated (600 + 600 cGy, at least 3 h apart) in a Shepherd Mark 1 irradiator. RBC-lysed BM nucleated cells (1 × 10^6^, unless otherwise indicated) were then injected retro-orbitally under isoflurane anaesthesia. For competitive reconstitution assays, 5 × 10^5^ competitor (BL/6-CD45.1) and 5 × 10^5^ control or *Maea*^*Csf1r-Cre*^ BM nucleated cells were mixed before injection.

### Colony-forming assays

For CFU-C assays, 100–500 sorted LSKs were plated in MethoCult^TM^ M3434 (Stem Cell Technologies) and colonies were scored on day 11 of culture.

### Homing assay

BM nucleated cells (10^7^ cells) from control and *Maea*^*Csf1r-Cre*^ mice (CD45.2) were transplanted into lethally irradiated BL/6-CD45.1 recipient mice. Recipients were sacrificed at 3 h post transplantation and femurs were processed for FACS analysis.

### RNA-seq analysis

Total RNA from 2000 sorted HSCs and LMPPs from BM of *Maea*^*Csf1r-Cre*^ young adults at 7–12 weeks of age was extracted using the RNAeasy Plus Micro kit (Qiagen), and assessed for integrity and purity using an Aligent 2100 Bioanalyzer (Agilent Technologies). Complementary DNA libraries were then generated using the SMART-Seq v4 Ultra Low Input RNA Kit for Sequencing (Clontech Laboratories) and the Nextera XT DNA Sample preparation Kit (Illumina). The libraries were then submitted for Illumina NextSeq 500 sequencing (150 bp single ended) according to the standard procedures. RNA-Seq data were then processed the following pipeline. Briefly, single-ended sequencing reads were aligned to the mouse genome (mm10) using Spliced Transcripts Alignment to a Reference (STAR 2.5.3a) method. Aligned reads were then quantified to annotation model (Ensembl Transcripts release 83) and normalised using Partek Flow. Raw and processed data generated from this RNA-seq analysis have been deposited in the Gene Expression Omnibus under accession number GSE133431. GSEA^48,49^ was then performed to identify differentially regulated pathways or to evaluate the overall enrichment of previously published gene sets.

### In vivo treatment

Poly I:C (Invivogen) was administered intraperitoneally (i.p.) every other day at 5 mg/kg for three doses for *Mx1*-Cre induction. NAC (Sigma #A7250) was administered at 50 mg/kg in PBS i.p. daily and supplemented at 1 mg/ml via drinking water which was refreshed twice a week. CFZ (CFZ/PR-171, Selleckchem), rapamycin (sirolimus, Selleckchem) and verapamil (Sigma #V4629) were dissolved in ethanol first and then diluted in 1% PEG400/1%Tween80/PBS for injections. CFZ was injected intravenously at 2 mg/kg on days 1 and 2 of each week. Rapamycin was administered at 4 mg/kg i.p. daily and supplemented at 15 ng/ml in drinking water when specified^50^. RTKi PKC412 (Midostaurin) was purchased from Selleck Chemicals (Cat# S8064) and injected i.p. daily at 3 mg/kg. LiCl^40^ (1 mEq/kg, Fisher #L121) and Verapamil^41^ (25 mg/kg, Sigma #V4629) were also injected i.p. daily. All drugs used for rescue purpose were administered starting the day after the pIpC induction and throughout the course until the day of analysis.

### Ubiquitin array

Lineage+ cells were depleted from isolated BMNCs using LD columns (Miltenyi Biotec #130-042-901) according to manufacturer’s instructions. The resulted lineage-BMNCs were immediately lysed at 1 × 10^7^ cells/ml Lysis Buffer from the Proteome Profiler Human Ubiquitin Array kit (R&D Systems, Cat#ARY027) and processed following manufacturer’s manual. Cells lysate from 2 to 4 mice were pooled to achieve ~0.2 mg protein/array. Protein of interests were pulled down from the cell lysate by the antibodies dotted on the provided membrane and then an HRP-conjugated Ubiquitin antibody was applied to detect the ubiquitin levels of the bound proteins. After chemiluminescent detection of ubiquitinated proteins, profiles of mean spot pixel density, corresponding to the level of ubiquitination of the captured protein, were generated using a transmission-mode scanner and the ‘Gel Analyzer’ function of ImageJ (version 1.52 NIH). Averaged replicate spot intensity from each array was analysed as a biological replicate.

### Half-life measurements of receptors and signalling molecules

To measure the half-life of surface receptors, BM cells were incubated in StemSpan SFEM (Stem Cell Technology #09650) with 50 μM cycloheximide (Millipore Sigma #508739) at 37 °C. Fractions of cells taken at indicated times of incubation were cooled and washed with FACS buffer containing protease and phosphatase inhibitors (Thermo Fisher #78442) immediately on ice. Expression of cell surface receptors was evaluated together with HSC markers by flow cytometry. Time zero corresponds to 30 min after start of the incubation to allow newly synthesised receptor proteins to reach the cell surface. To examine whether autophagy inducers could normalise receptor half-life, the same assay was done in the presence of 10 mM LiCl^40^.

### Ubiquitination assay and immunoblotting

For analysis of ubiquitination of FLT3 by MAEA, 10^7^ cells 293T (ATCC) cells were seeded in 10 cm dish in DMEM (Corning) supplemented with 10% FBS, L-glutamine and antibiotics (Invitrogen) 24 h before transfection with 2 μg of the following constructs using Lipofectamine 2000 according to the manufacturer’s instructions (Invitrogen): pLenti-puro HA-Ubiquitin was a gift from Melina Fan (Addgene plasmid #74218; http://n2t.net/addgene:74218; RRID:Addgene_74218). pLenti CMV rtTA3 Blast (w756-1) was a gift from Eric Campeau (Addgene plasmid #26429; http://n2t.net/addgene:26429; RRID: Addgene_26429). pLenti-puro-FLAG-MAEA and pLenti-puro- MAEA-FLAG were constructed by amplifying the MAEA CDS (GenBank Accession#: BC001225) with FLAG tag at either N-terminal or C-terminal and cloned into BamHI and EcoRI sites of pLenti-puro. pLenti-puro was a gift from Ie-Ming Shih (Addgene plasmid #39481; http://n2t.net/addgene:39481; RRID:Addgene_39481). Cells were then harvested in ice-cold PBS buffer (Corning), pelleted, and lysed in RIPA buffer (Thermo Scientific) containing complete protease inhibitor (Thermo Scientific) and phosphatase inhibitors (Thermo Scientific). Protein concentration was determined by the BCA Kit (Pierce). Whole cell lysates in RIPA buffer aforementioned (1000 µg) were incubated with anti-HA agarose overnight, then washed three times with RIPA buffer with protease and phosphatase inhibitors, and subjected electrophoresis and immunoblot analysis. Equal amounts of whole cell lysate (20 μg) were also subjected electrophoresis and immunoblot analysis. Antibodies used for immunoblotting analysis are as follows: Anti-FLT3 (PA5-34448, Thermo Fisher Scientific), anti-HA-Tag (C29F4, Cell Signalling Technology), anti-MAEA (I-20, Santa Cruz technology), anti-Flag (F1804, Sigma). For immunoprecipitations of tagged proteins, we used: Anti-HA − Agarose (A2095, Sigma). MG132 (M7449, Sigma).

### Autophagy detection by flow cytometry

BM or sorted LSK cells were cytokine-starved in StemSpan or nutrient starved in EBSS (Gibco #14155063) at 37 °C in 5% CO_2_ for 3 h in the presence or absence of lysosome inhibitors 100 μM leupeptin (Sigma #L8511) + 20 mM ammonium chloride (freshly prepared) for autophagy flux measurement. Rescue experiments were done in the presence of 0.2 μM rapamycin or 10 mM LiCl. At the end of the starvation culture, cells were collected by centrifugation and stained for cell surface markers followed by LC3-II staining using a FlowCellect Autophagy LC3 Antibody-based Assay Kit (#FCCH100171, EMD Millipore, MA, USA) following manufacturer’s instructions. A membrane permeabilization step in this kit allows the extraction of non-membrane bound LC3-I and thus detection of LC3-II by flow cytometry^51^.

### Immunofluorescence imaging

BM cells were processed similarly as for flow cytometry for cell surface and intracellular marker staining. Desired population of cells were then sorted into DAPI/PBS, concentrated by centrifugation and transferred onto Poly L-lysine-coated slides (Sigma #P0425). After the cells were settled, the slides were then mounted following standard procedures for image acquisition on a ZEISS Axio examiner D1 microscope with a confocal scanner unit, CSUX1CU (Yokogawa). For colocalization analysis of FLT3, MPL and LC3, freshly isolated or starved BM cells were stained for surface markers and LC3 as described above. LSKs were then sorted onto slides for confocal immunofluorescence analysis. Random fields of cells were imaged, and FLT3^+^ or MPL^+^ and LC3^+^ double-positive foci were manually quantified in Slidebook for each cell in Slidebook 6.0 software.

### Electron microscopy and morphometric analysis of the autophagic compartments

For electron microscopy analysis, freshly sorted HSCs were pelleted and fixed in 2.5% glutaraldehyde in 0.1 M sodium cacodylate at room temperature for at least a half hour, and stored at 4 °C in fixative until embedding. The cells were then post-fixed 1:1 with 2% Osmium Tetroxide in 0.2 M Cacodylate buffer and spun gain to form a visible pellet. Cells were further fixed with 2% Uranyl acetate (aq), dehydrated through a graded series of ethanol and embedded in LX112 resin (LADD Research Industries, Burlington VT). Ultrathin sections were cut on a Leica UC7, stained with uranyl acetate, followed by lead citrate and viewed on a JEOL 1200EX transmission electron microscope at 80 kv. Autophagic vacuoles were identified using previously established criteria^52,53^ by quantification by experts (S.D. and A.M.C) blind to the genotypes. Autophagic vacuoles (vesicles, 0.5 μm) were classified as APG when they met two or more of the following criteria: double membranes (complete or at least partially visible), absence of ribosomes attached to the cytosolic side of the membrane, luminal density similar to cytosol and identifiable organelles or regions of organelles in their lumen. Vesicles of similar size but with a single membrane (or <40% of the membrane visible as double), luminal density lower than the surrounding cytosol or multiple single membrane-limited vesicles containing light or dense amorphous material were classified as autophagolysosomes (AUT).

### Statistics and reproducibility

In each experiment, each mouse was analysed as a biological replicate. All data shown are derived from at least two independent experiments with similar results. Data visualisation (shown as mean ± sem) and statistical analysis were performed using Graphpad Prism 7. Unpaired two-sided Student’s *t* test was used to assess statistical significance when comparing two samples unless otherwise indicated.

### Reporting summary

Further information on research design is available in the Nature Research Reporting Summary linked to this article.

## Supplementary information

Supplementary Information

Reporting Summary

## Data Availability

There are no restrictions on data availability in this manuscript. All the information is included in the manuscript. Raw and processed reads data from the RNA-seq have been deposited in the Gene Expression Omnibus under accession number GSE133431. Other raw data or images can be made available upon reasonable request. Source data are provided with this paper.

## Source data

Source Data
