# Supplementary material for: MAEA is an E3 ubiquitin ligase promoting autophagy and maintenance of haematopoietic stem cells
Source: Nat Commun. 2021 May 4;12:2522. doi: 10.1038/s41467-021-22749-1 (PMC8097058; doi:10.1038/s41467-021-22749-1)
Supplement: Supplementary file 1 — Supplementary Information [file 41467_2021_22749_MOESM1_ESM.pdf]

Supplementary Fig. 1

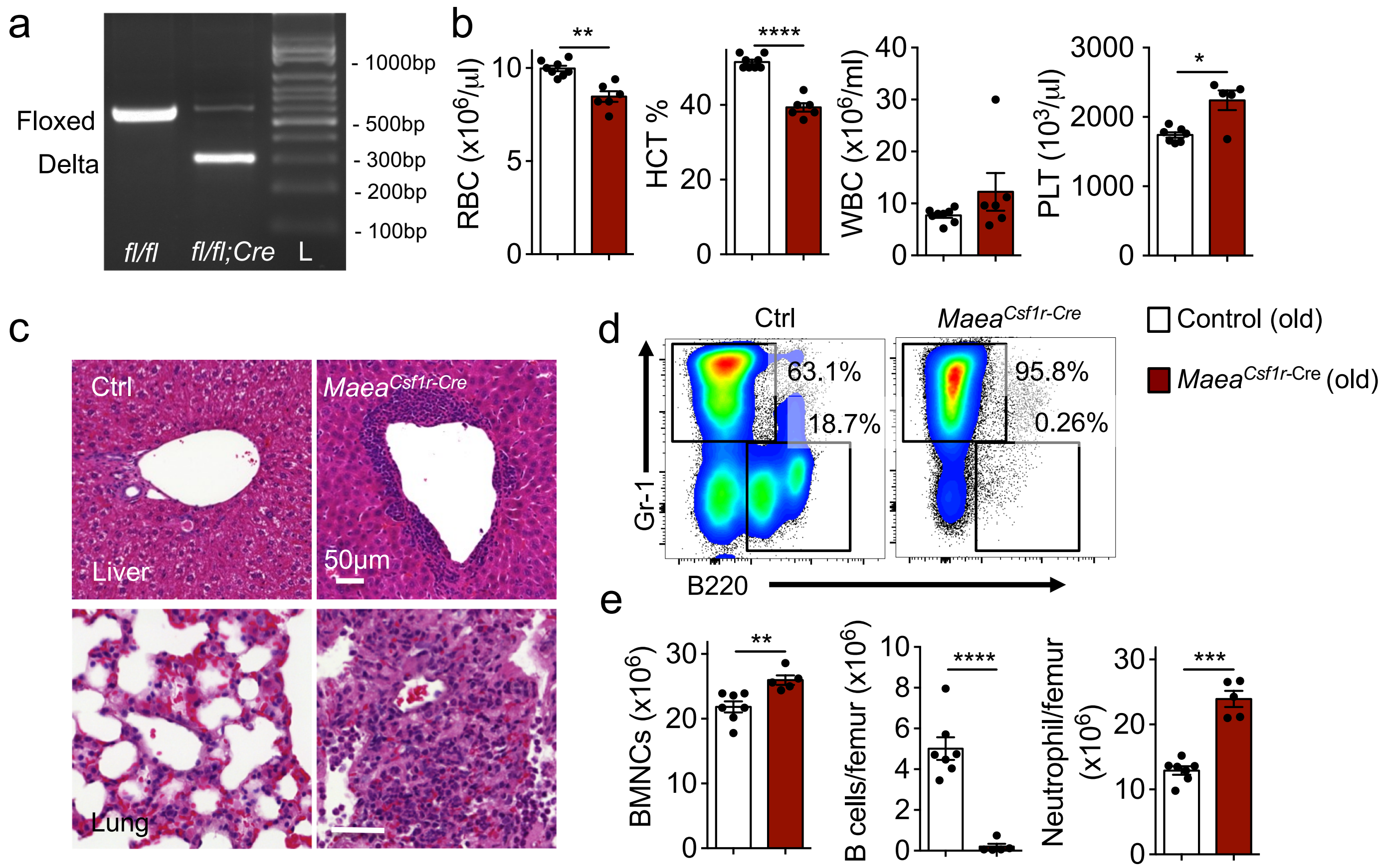

Supplementary Fig. 1. *Maea*$^{Csf1r\text{-}Cre}$ mice develop myeloproliferation and lymphopenia. **a**, PCR on genomic DNA isolated from BM HSCs of *Maea*^{floxed} *(fl/fl)* control and *Maea*$^{Csf1r\text{-}Cre}$ (fl/fl;Cre+) mice using primers listed in Supplementary Table 1. L=100bp DNA ladder. This experiment has been performed routinely on all mice used in this and previously published (ref.15) study. **b**. Peripheral blood indices of 7 months old control (n=8) and *Maea*$^{Csf1r\text{-}Cre}$ (n=6) mice. RBC, red blood cells; HCT, haematocrit; WBC, white blood cells; PLT, platelets. RBC p=0.0018, HCT p<0.0001, PLT p=0.022. **c**, Representative H&E stained paraffin sections from the livers and lungs of 7 months old control (n=8) and *Maea*$^{Csf1r\text{-}Cre}$ (n=6) mice shown in Fig. 1c. Scale bar=50µm. **d** and **e**, Representative FACS plots (d) and quantification (e) of BM cellularity, Gr-1$^+$ neutrophils and B220$^+$ B cells in total BM nucleated cells in 7 months old control (n=7) and *Maea*$^{Csf1r\text{-}Cre}$ (n=6) mice. BMNC p=0.0042, B cell p<0.0001, Neu p=0.0002. Data are shown as mean ± s.e.m. *p< 0.05, **p< 0.01, ***p< 0.001, ****p<0.0001 by unpaired two-sided Student's t test.

Supplementary Fig. 2

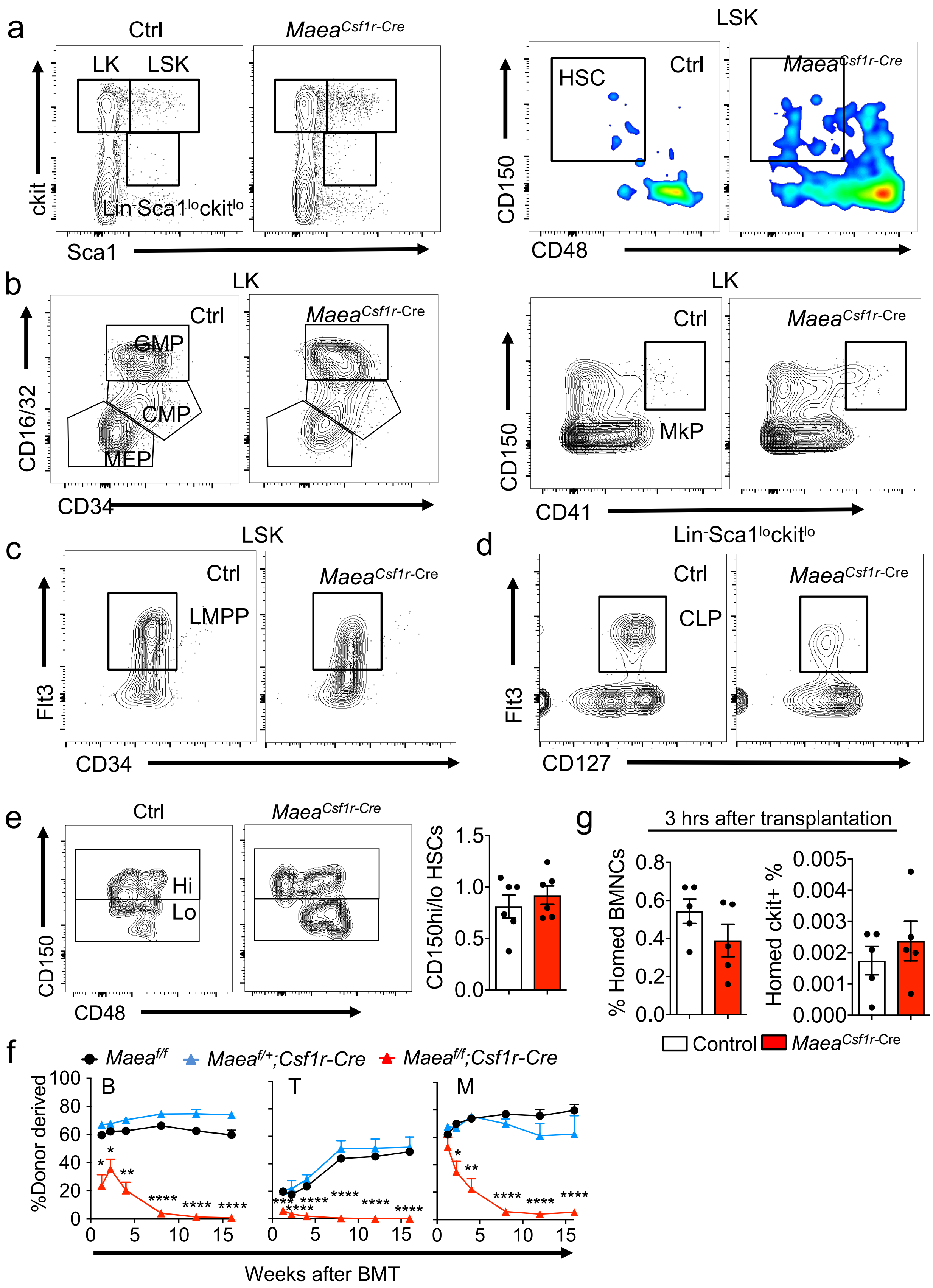

Supplementary Fig. 2. Altered hematopoiesis in *Maea*$^{Csf1r\text{-}Cre}$ mice. a, Representative FACS plots and quantifications showing increased LSK, LK and HSCs in the *Maea*$^{Csf1r\text{-}Cre}$ mice. Events are pre-gated on the lineage- populations. **b,** Representative gating strategy for GMP, CMP, MEP and MkP from the LK population in a. **c,** Representative FACS plots showing decreased frequencies of LMPPs in the LSK population from the *Maea*$^{Csf1r\text{-}Cre}$ mice. **d,** Representative FACS plots showing decreased frequencies of CLPs in the $Lin^{-}Sca1^{lo}ckit^{lo}$ population from the *Maea*$^{Csf1r\text{-}Cre}$ mice. LMPP: lymphoid-primed multipotent progenitors; CMP: common myeloid progenitors; CLP: common lymphoid progenitors; GMP: granulocyte-macrophage progenitors; MEP: megakaryocyte-erythrocyte progenitors. **e**, Quantification of CD150 high vs low myeloid-biased vs lymphoid competent HSCs in *Maea*$^{Csf1r\text{-}Cre}$ mice (n=6). **f**, Peripheral blood donor chimaerism at indicated time points after competitive BM transplantation of equal number ($1x10^6$) of CD45.1 wild type competitor BM cells and CD45.2 donor BM cells from indicated genotypes into lethally irradiated CD45.1 wild type recipients (fl/fl: n=4, fl/+; Cre: n=5; fl/fl; Cre: n=6). Statistic significance determined by multiple unpaired *t* test with Holm-Sidak's corrections for multiple comparisons. B 1.25 week p=0.015, 2.25 week p=0.0377, 4 week p=0.00148, 8, 12 16 weeks p<0.0001. T 1.25 week p=0.0003, 2.25~16 weeks p<0.0001. M 1.25 week p=0.422, 2.25 week p=0.0175, 4 week p=0.0027, 8, 12 16 weeks p<0.0001. **g**, Homed control and *Maea*$^{Csf1r\text{-}Cre}$ total BM cells and ckit+ cells in recipient BM 3 hours after transplantation (n=5 each group). All data are shown as mean ± s.e.m. **p< 0.01, ***p< 0.001, ****p<0.0001 by unpaired two-sided Student's *t* test unless otherwise indicated.

Supplementary Fig.3

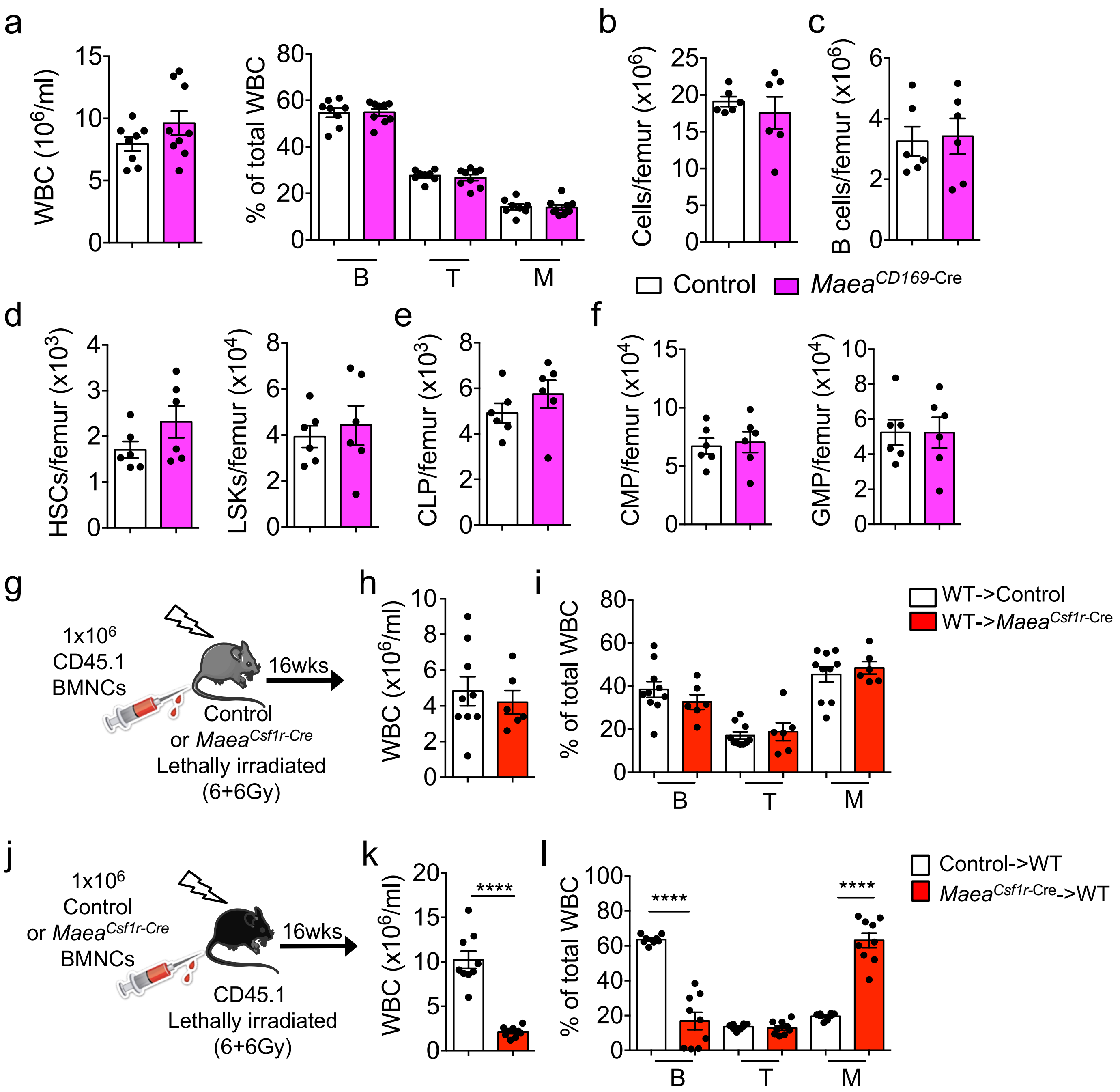

Supplementary Fig.3 Lymphopenia in *Maea*$^{Csf1r\text{-}Cre}$ mice is caused by hematopoietic cell-intrinsic defects but not by macrophages. **a,** Analysis of white blood cells (WBCs) in *Maea*$^{CD169\text{-}Cre}$ mice (n=8). p=0.1493 **b,** Quantification of *Maea*$^{CD169\text{-}Cre}$ BM cellularity (n=6). p= 0.5258. **c,** Total B cell numbers in the BM of *Maea*$^{CD169\text{-}Cre}$ mice (n=6). p=0.4379. **d-f,** Quantification of HSPC numbers (d) HSC p=0.1549, LSK p=0.1037, lymphoid progenitors (e) p=0.2758 and myeloid progenitors (f) CMP p=0.5874, GMP p=0.6759 in *Maea*$^{CD169\text{-}Cre}$ mice (n=6). **g,** Experimental design to examine the contribution of the microenvironment to the lymphoid defects in the *Maea*$^{Csf1r\text{-}Cre}$ mice. **h,** Quantification of leukocytes in peripheral blood of control (n=8) and *Maea*$^{Csf1r\text{-}Cre}$ (n=6) mice reconstituted with wild-type BM cells. p=0.6267. **i,** Frequencies of B, T, and myeloid cells in peripheral blood of control (n=8) and *Maea*$^{Csf1r\text{-}Cre}$ (n=6) mice reconstituted with wild-type BM cells. B p=0.3468, T p=0.5762, M p=0.6363. **j,** Experimental design to examine the contribution of the hematopoietic cells to the lymphoid defects in the *Maea*$^{Csf1r\text{-}Cre}$ mice. **k,** Quantification of leukocytes in peripheral blood of wild-type mice reconstituted with control and *Maea*$^{Csf1r\text{-}Cre}$ BM cells (n=9 each over 2 independent experiments). p<0.0001. **l,** Frequencies of B, T, and myeloid cells in peripheral blood of wild-type mice reconstituted with control and *Maea*$^{Csf1r\text{-}Cre}$ BM cells (n=9 each over 2 independent experiments). B and M p<0.0001, T p=0.8259. Data are shown as mean ± s.e.m. ****p<0.0001 by unpaired two-sided Student's *t* test.

Supplementary Fig.4

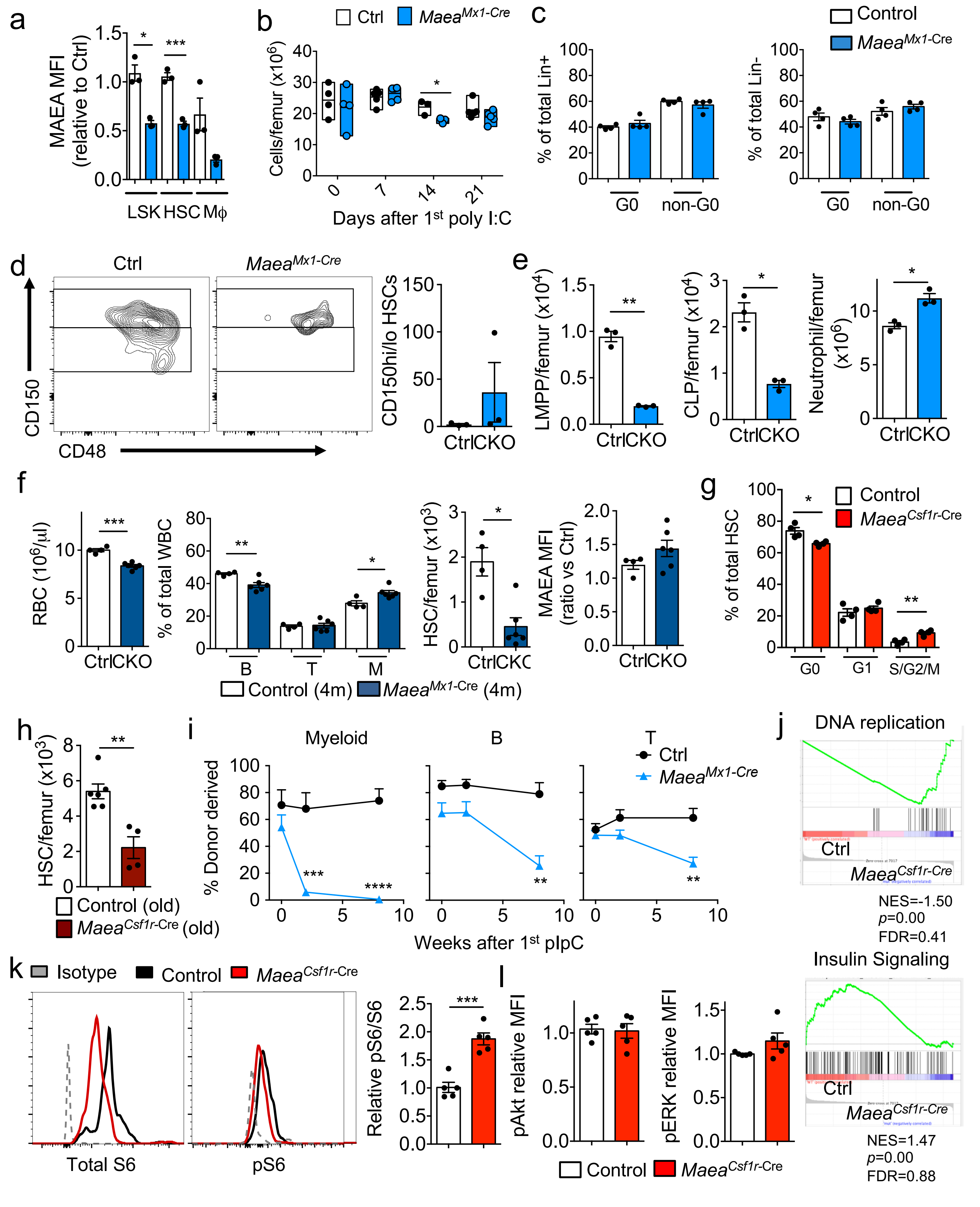

Supplementary Fig.4 *Maea*-deletion impairs HSC quiescence and function. a, Maea expression in control and $Maea^{Mx1\text{-}Cre}$ BM cells at 3wks after 1st poly I:C injection (n=3 animals). LSK *p*=0.0168, HSC *p*=0.0009, M *p*=0.1098. **b,** BM cellularity of control and $Maea^{Mx1\text{-}Cre}$ mice at indicated time points after 1st poly I:C injection (n=4 each group). Data are represented as floating boxes with boundaries indicate minima to maxima of each dataset and middle line indicates the mean. *p*=0.0235. **c,** Cell cycle profiles of control and $Maea^{Mx1\text{-}Cre}$ total lineage+ and lineage- cells at 21days after 1st poly I:C injection (n=4 each group). **d**, Representative FACS plots and quantification of CD150 high vs low myeloid-biased vs lymphoid competent HSCs in BM of $Maea^{Mx1\text{-}Cre}$ mice 14days after 1st poly I:C injection (n=3 each group). **e**, Quantification of lymphoid progenitor cells and neutrophils in BM of $Maea^{Mx1\text{-}Cre}$ mice 14days after 1st poly I:C injection (n=3). *p* value from left to right are 0.0035, 0.0135, 0.022. **f,** Analysis of red blood cell (RBC) counts, white blood cell (WBC), HSC numbers and MAEA expression in control and $Maea^{Mx1\text{-}Cre}$ mice 4 months after pIpC injections (n=4 and 6). *p* value from left to right are 0.0001, 0.0046, 0.0174, 0.0104, 0.899. **g,** Cell cycle analysis of BM HSCs from control and $Maea^{Csf1r\text{-}Cre}$ mice by Ki-67 and H33342 dye staining (n=4). *p* value from left to right are 0.035, 0.0098. **h,** Quantification of HSC numbers in 7 months old control and $Maea^{Csf1r\text{-}Cre}$ BM (n=6 and 4 respectively). *p*=0.0016. **i,** Donor chimaerism in peripheral blood myeloid, B and T lineage leukocytes from control and $Maea^{Mx1\text{-}Cre}$ mixed chimeric mice at indicated time points after 1st poly I:C injection (n=10 over 2 independent experiments). *p* value from left to right are 0.0004, 0.00009, 0.0058, 0.0036. **j,** GSEA enrichment plot showing significant up-regulation of DNA replication pathway and down-regulation of insulin signaling pathway in $Maea^{Csf1r\text{-}Cre}$ HSCs. **k**, Representative histograms showing total S6 and pS6 levels and pS6/S6 ratio in control and $Maea^{Csf1r\text{-}Cre}$ HSCs (n=5). **l**, pAKT (S473) and pERK1/2 (p44/42 MAPK, T202/Y204) levels in control and $Maea^{Csf1r\text{-}Cre}$ HSCs (n=5). *p* value from left to right are 0.7894, 0.5369. All data are shown as mean ± s.e.m. *$p < 0.05$, **$p < 0.01$, ***$p < 0.001$, ****$p < 0.0001$ by unpaired two-sided Student's *t* test unless otherwise indicated.

Supplementary Fig.5

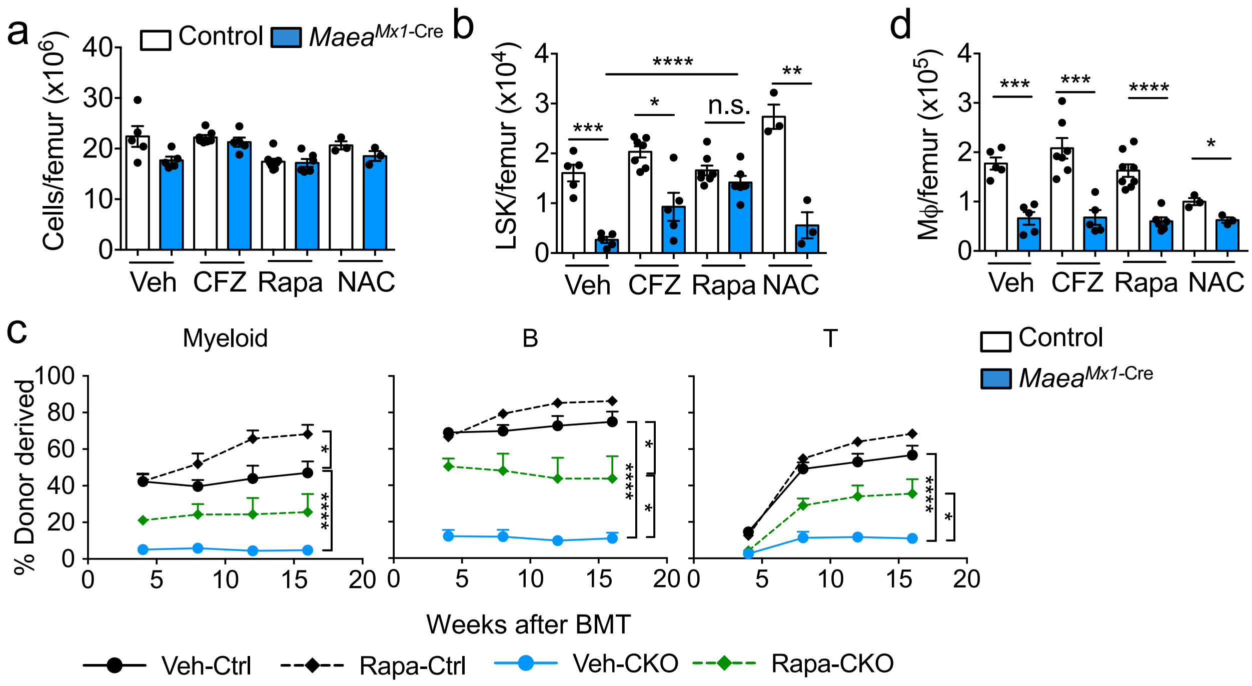

Supplementary Fig. 5. Deletion of *Maea* results in aberrant HSC activation and depletion in a mTOR-dependent manner. a and **b,** Quantification of BM cellularity (a) and BM LSK numbers (b) in control and $Maea^{Mx1\text{-}Cre}$ mice treated with vehicle, carfilzomib, rapamycin or NAC 3wks after poly I:C induction. **c**, Donor chimaerism in peripheral blood of CD45.1 lethally irradiated wild type recipients at indicated time points after competitive BM transplantation of equal number of CD45.1 wild type competitor BM cells and CD45.2 donor BM cells from indicated groups (n=5 each group). Statistic significance determined by multiple unpaired *t* test with Holm-Sidak's corrections for multiple comparisons. **d,** Quantification of BM macrophage numbers in control and $Maea^{Mx1\text{-}Cre}$ mice treated with vehicle, carfilzomib, rapamycin or NAC 3wks after poly I:C induction. Data are shown as mean ± s.e.m. *p* value from left to right are 0.0003, 0.0003, <0.0001, 0.0167. n.s., not significant. (a-b, and d: Veh: n=5, CFZ: n=6, Rapa: n=7, NAC: n=3 over 2 independent experiments). *$p < 0.05$, **$p < 0.01$, ***$p < 0.001$, ****$p < 0.0001$ by unpaired two-sided Student's *t* test unless otherwise indicated.

Supplementary Fig.6

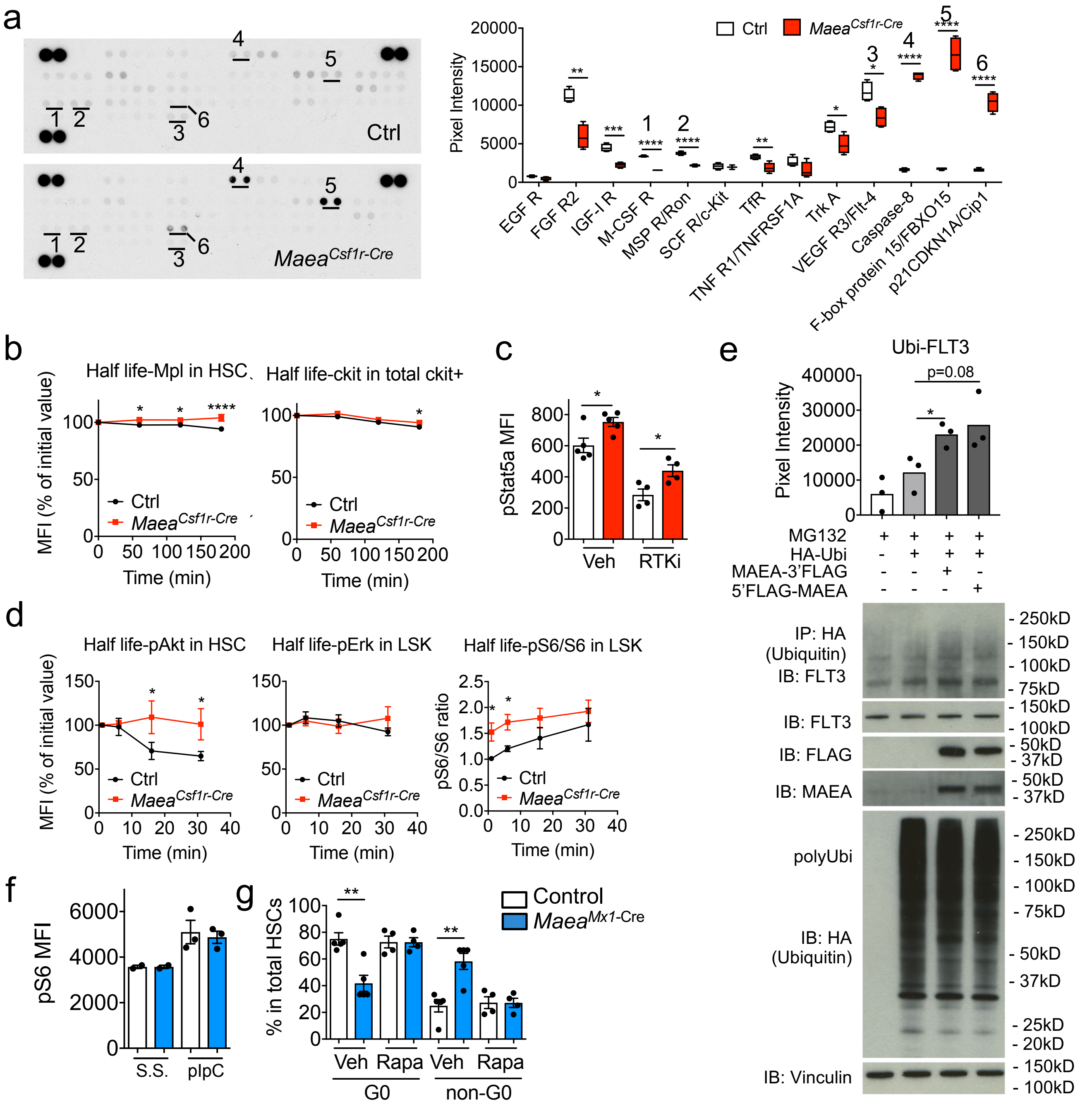

Supplementary Fig. 6. MAEA regulates receptor stability and signaling. a, Representative ubiquitin array images and quantified mean spot pixel intensity of selected targets from freshly isolated control (Ctrl) and *Maea*$^{Csf1r\text{-}Cre}$ lineage negative bone marrow (BM) cells (n=4 independent experiments using pooled animals of the same genotype). Data are represented as boxes-and-whiskers with the whiskers indicate minima to maxima of each dataset, the boxes extend from the 25th to 75th percentiles, and the middle line indicates the mean. **b,** FACS evaluation of surface receptor Mpl and ckit half-lives in control and *Maea*$^{Csf1r\text{-}Cre}$ HSPCs incubated in the presence of 50μM cycloheximide (n=4). *p* value from left to right are 0.0138, 0.0196, <0.0001 and 0. 0449. **c**, Phospho-flow evaluation of pSTAT5a in control and *Maea*$^{Csf1r\text{-}Cre}$ LSKs from mice treated with vehicle (n=5) or RTKi PKC412 (n=4). *p* value from left to right are 0.0301, 0.0265. **d,** Phospho-flow evaluation of signaling molecules pAkt, pErk and pS6/S6 ratio in control and *Maea*$^{Csf1r\text{-}Cre}$ HSPCs at indicated time points after cytokine (TPO, FLT3 and SCF 20ng/ml each) stimulation (pAkt and pErk: n=4, pS6/S6 n=5). *p* value from left to right are 0.0102, 0.0221, 0.0143, 0.0142. In a and c, data are shown as mean ± s.e.m. *p< 0.05 and ****p<0.0001 by two-way ANOVA multiple comparisons. **e,** Ubiquitination of FLT3 by MAEA shown by immunoprecipitation of HA–Ubiquitin in 293T cells transfected with constructs expressing HA-Ubiquitin, MAEA-3'FLAG and 5'FLAG-MAEA and cultured in the presence of 10 μM MG132 for 4 hours prior to harvest 24 hours post transfection. The whole cell lysates and immunoprecipitates were analysed by the indicated immunoblots. Ubiquitinated FLT3 was quantified using the gel analyzer tool in ImageJ. Data are shown as mean ± s.e.m. *p< 0.05 by Welch's t test. IB, immunoblotting. IP, immunoprecipitation (n=3 independent experiments). *p=0.0485. **f,** pS6 staining in BM HSCs isolated from control (n=5) and *Maea*$^{Mx1\text{-}Cre}$ (n=5) mice without pIpC injection (steady state, S.S.) and 1 day after the last pIpC injection. **g,** Cell cycle profile, by Ki67 and H33342 staining, of BM HSCs isolated from control and *Maea*$^{Mx1\text{-}Cre}$ mice treated with vehicle (n=5) or rapamycin (Rapa) (n=4) for 3 weeks after pIpC. p=0.0026. Data are shown as mean ± s.e.m. *p< 0.05, **p< 0.01, ****p<0.0001 by Welch's two-sided *t* test unless otherwise indicated.

Supplementary Fig.7

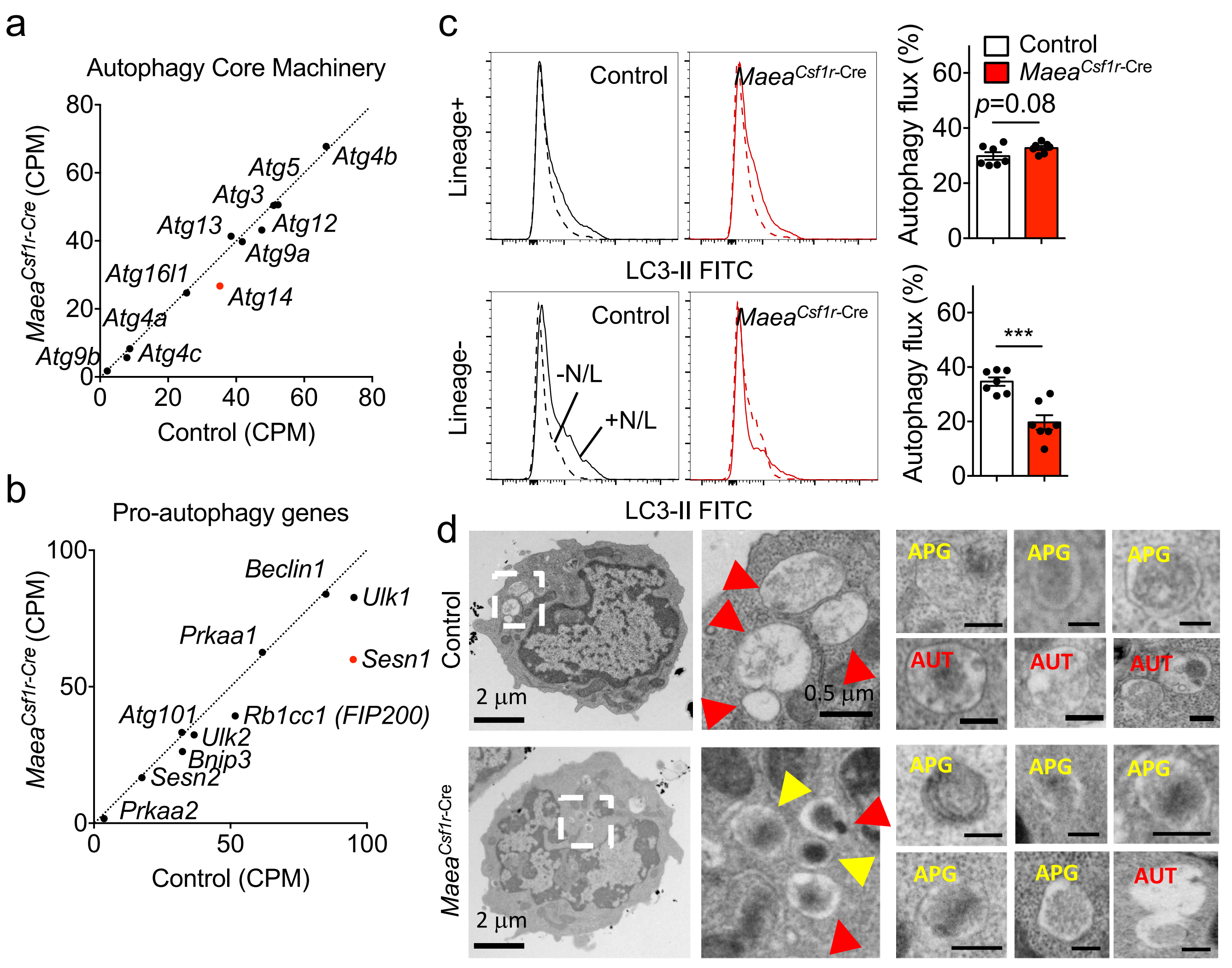

Supplementary Fig.7. Normal expression of autophagy machinery but impaired autophagy flux in *Maea*$^{Csf1r\text{-}Cre}$ HSCs. a, Expression of core autophagy machinery in control and *Maea*$^{Csf1r\text{-}Cre}$ HSCs from RNA-seq analysis. **b**, Expression of pro-autophagy genes in control and *Maea*$^{Csf1r\text{-}Cre}$ HSCs from RNA-seq analysis. **c**, Representative histograms and FACS quantifications showing autophagy flux by LC3-II in control and *Maea*$^{Csf1r\text{-}Cre}$ lineage+ (upper) and lineage- (lower) BM cells, measured at the same time as in Fig. 4c (n=7). Data are shown as mean ± s.e.m. ***p=0.0008 by unpaired two-sided Student's *t* test. **d**, Additional micrographs of whole cells (left) and examples of autophagosomes (APG, yellow arrows) and autolysosomes (AUT, red arrows) from control and *Maea*$^{Csf1r\text{-}Cre}$ HSCs as shown in Fig. 4b-c. Bars in inserts = 0.1mm. Analysis were applied to cells blindly from two independent experiments. Unblinding was done during data plotting.

Supplementary Fig.8

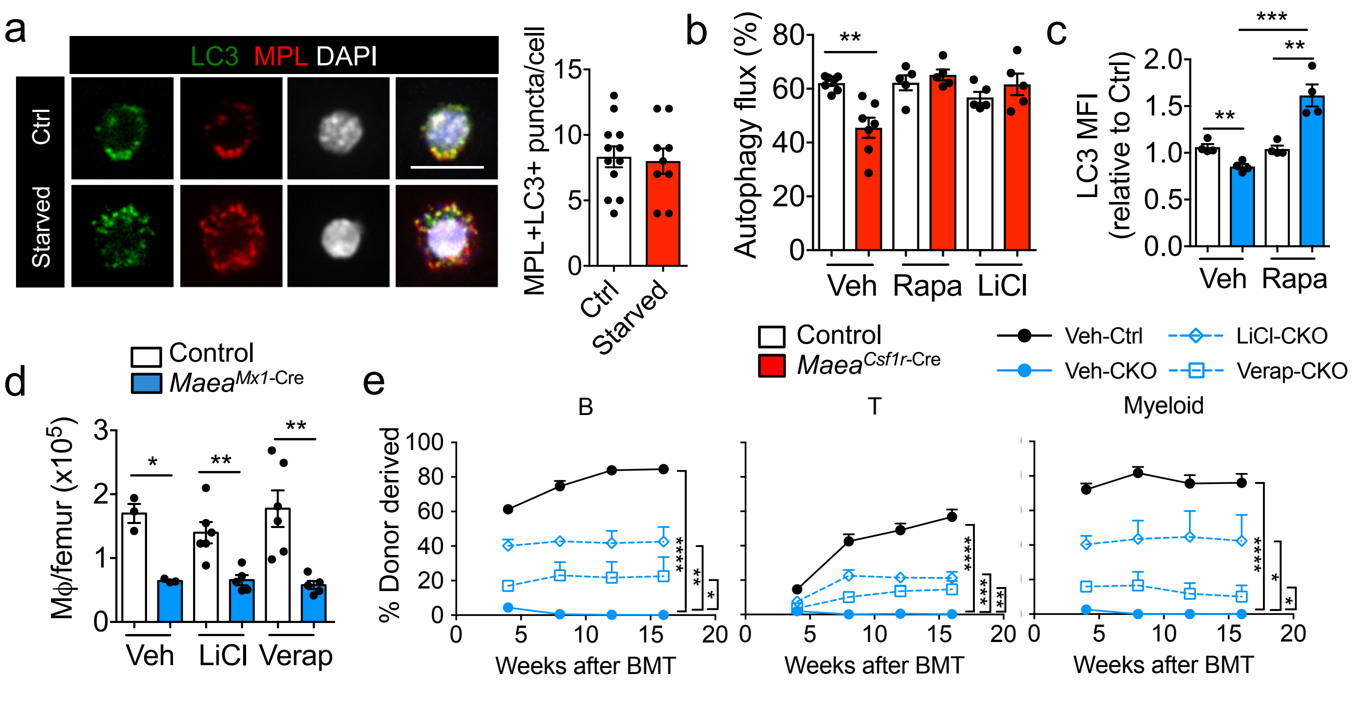

Supplementary Fig.8. MAEA regulates receptor stability and HSC maintenance via autophagy. a, Representative immunofluorescence images and quantification showing subcellular colocalization of MPL and LC3 in freshly isolated (Ctrl) and starved (cultured ex vivo in StemSpan with no cytokines but in the presence of lysosome inhibitors N/L for 3hrs to induce autophagy) HSPCs (n=12 and 9 cells analyzed respectively). Scale bar=10μm. **b**, Evaluation of autophagy flux in control and *Maea*$^{Csf1r\text{-}Cre}$ HSCs incubated with 0.2μM rapamycin and 10mM LiCl (n=5 each group). *p* value from left to right are 0.0038, 0.7895, 0.5365. **c**, LC3-II staining in BM HSCs isolated from control and *Maea*$^{Mx1\text{-}Cre}$ mice treated with vehicle or rapamycin (Rapa) for 3 weeks after pIpC (n=4 each group). Statistic significance determined by 2-way ANOVA multiple comparisons. *p* value from left to right are 0.0013, 0.0001, 0.0010. **d**, Quantification of BM macrophages in control and *Maea*$^{Mx1\text{-}Cre}$ mice treated with vehicle (Veh), LiCl, or verapamil (Verap) 3wks after poly I:C induction (Veh: n=3, LiCl: n=6, Verap: n=6). *p* value from left to right are 0.0183, 0.005, 0.0078. **e**, Donor chimaerism in peripheral blood of CD45.1 lethally irradiated wild type recipients at indicated time points after competitive BM transplantation of equal number of CD45.1 wild type competitor BM cells and CD45.2 donor BM cells from indicated groups (n=5 each group). Statistic significance determined by multiple unpaired *t* test with Holm-Sidak's corrections for multiple comparisons. All data are shown as mean ± s.e.m. *$p < 0.05$, **$p < 0.01$, ***$p < 0.001$, ****$p < 0.0001$ by unpaired two-sided Student's *t* test unless otherwise indicated.

Supplementary Table 1

**Primer names**	**Primer sequences**
MaeaI4_F2	caccagctcaggcagttaca
Maea3loxP_R2	aactgatggcgagctcaga
MaeaI4_R4	cgggaagaagtgggattacc
