# Supplementary material for: MAEA is an E3 ubiquitin ligase promoting autophagy and maintenance of haematopoietic stem cells
Source: Nat Commun. 2021 May 4;12:2522. doi: 10.1038/s41467-021-22749-1 (PMC8097058; doi:10.1038/s41467-021-22749-1)
Supplement: Supplementary file 2 — Reporting Summary [file 41467_2021_22749_MOESM2_ESM.pdf]

nature research

Corresponding author(s): *Double-blind peer review submissions: write DBPR and your manuscript number here instead of author names.*

Last updated by author(s): *YYYY-MM-DD*

Reporting Summary

Nature Research wishes to improve the reproducibility of the work that we publish. This form provides structure for consistency and transparency in reporting. For further information on Nature Research policies, see our Editorial Policies and the Editorial Policy Checklist.

Statistics

For all statistical analyses, confirm that the following items are present in the figure legend, table legend, main text, or Methods section.

n/a	Confirmed	
☐	☒	The exact sample size (*n*) for each experimental group/condition, given as a discrete number and unit of measurement
☐	☒	A statement on whether measurements were taken from distinct samples or whether the same sample was measured repeatedly
☐	☒	The statistical test(s) used AND whether they are one- or two-sided<br>*Only common tests should be described solely by name; describe more complex techniques in the Methods section.*
☐	☒	A description of all covariates tested
☐	☒	A description of any assumptions or corrections, such as tests of normality and adjustment for multiple comparisons
☐	☒	A full description of the statistical parameters including central tendency (e.g. means) or other basic estimates (e.g. regression coefficient) AND variation (e.g. standard deviation) or associated estimates of uncertainty (e.g. confidence intervals)
☐	☒	For null hypothesis testing, the test statistic (e.g. *F*, *t*, *r*) with confidence intervals, effect sizes, degrees of freedom and *P* value noted<br>*Give P values as exact values whenever suitable.*
☒	☐	For Bayesian analysis, information on the choice of priors and Markov chain Monte Carlo settings
☐	☒	For hierarchical and complex designs, identification of the appropriate level for tests and full reporting of outcomes
☒	☐	Estimates of effect sizes (e.g. Cohen's *d*, Pearson's *r*), indicating how they were calculated

Our web collection on statistics for biologists contains articles on many of the points above.

Software and code

Policy information about availability of computer code

Data collection	BD FACSDIVA™ Software (V4.1, BD Biosciences) was used for collection of flow cytometry data.<br>Confocal images were acquired using Slidebook 6.0 (3i).
Data analysis	Immunofluorescence images were analyzed using Image J ((1.52v NIH) https://imagej.nih.gov/ij/) and Slidebook 6.0 (3i).<br>Immunoblotting images were quantified using the "Gel Analyzer" function of Image J.<br>Flow cytometry analysis was performed using FlowJo 10.4.0 (LLC).<br>RNA-seq analysis was performed using Partek Flow Genomic analysis software (https://www.partek.com/partek-flow/) and Gene Set Enrichment Analysis (GSEA) (https://www.gsea-msigdb.org/gsea/index.jsp).<br>All graphical plots were made using GraphPad Prism software 7.04 (GraphPad) https://www.graphpad.com

For manuscripts utilizing custom algorithms or software that are central to the research but not yet described in published literature, software must be made available to editors and reviewers. We strongly encourage code deposition in a community repository (e.g. GitHub). See the Nature Research guidelines for submitting code & software for further information.

Data

Policy information about availability of data

All manuscripts must include a data availability statement. This statement should provide the following information, where applicable:

- Accession codes, unique identifiers, or web links for publicly available datasets
- A list of figures that have associated raw data
- A description of any restrictions on data availability

There are no restrictions on data availability in this manuscript. All the information is included in the manuscript. Raw and processed reads data from the RNA-seq have been deposited in the Gene Expression Omnibus under accession number GSE133431. Other raw data or images can be made available upon reasonable

request.

Field-specific reporting

Please select the one below that is the best fit for your research. If you are not sure, read the appropriate sections before making your selection.

☒ Life sciences ☐ Behavioural & social sciences ☐ Ecological, evolutionary & environmental sciences

For a reference copy of the document with all sections, see nature.com/documents/nr-reporting-summary-flat.pdf

Life sciences study design

All studies must disclose on these points even when the disclosure is negative.

Sample size	Sample sizes were determined by data distribution, technical variability and changes in treatment conditions. In all instances we indicate what "n" refers to and state the number of individual experiments. The number of animals used for experiment was calculated through power analysis based on previous results.
Data exclusions	No data points were excluded in this study.
Replication	Data comes from a minimum of three independent experiments unless otherwise stated. Each data point is an individual mouse or a pool of several mice from the same genotype unless otherwise indicated.
Randomization	Animals and samples were randomized during in vivo treatment, processing and data collection.
Blinding	Investigators were blinded to the genotype or treatment during sample processing, data collection and image analysis.

Reporting for specific materials, systems and methods

We require information from authors about some types of materials, experimental systems and methods used in many studies. Here, indicate whether each material, system or method listed is relevant to your study. If you are not sure if a list item applies to your research, read the appropriate section before selecting a response.

Materials & experimental systems

n/a	Involved in the study	
☐	☒	Antibodies
☒	☐	Eukaryotic cell lines
☒	☐	Palaeontology and archaeology
☐	☒	Animals and other organisms
☒	☐	Human research participants
☒	☐	Clinical data
☒	☐	Dual use research of concern

Methods

n/a	Involved in the study	
☒	☐	ChIP-seq
☐	☒	Flow cytometry
☒	☐	MRI-based neuroimaging

Antibodies

Antibodies used

Purified goat anti-MAEA polyclonal antibody (I-20) was purchased from Santa Cruz (discontinued) and used at 1:100 concentration. Conjugated donkey anti-goat IgG secondary antibodies were from ThermoFisher (A21447) and used at 1:800 concentration. An anti-MAEA monoclonal antibody (92.25) was generated by our laboratory and recently described(ref.15).

Fluorochrome-conjugated or biotinylated antibodies against mouse F4/80-PE (BM8; 123110), CD115-PE/C7 (AFS98; 25-1152-82), B220-APC-eFluor780 (RA3-6B2; 47-0452-82), anti-CD3e-PerCP-Cy5.5 (145-2C11; 45-0031-82), Gr-1(Ly6C/G)-FITC (RB6-8C5; 11-5931-85), CD11b-PE (M1/17; 12-0112-83), CD45.1-PE/Cy (A20; 25-0453-82), CD45.2-FITC (104; 109806), F4/80 (clone BM8) , CD45 (clone 30-F11), c-kit/CD117-PE/Cy7 (2B8; 105814), Sca1-FITC (clone D7, 11-5981-85), CD150-PE (clone TC15-12F12.2, 115904), CD48-PerCP-eFluor710 (HM48-1; 46-0481-85), CD16/32- APC/CY7 (clone 93, 101328), CD34-eFlour660 (clone RAM34, 50-0341-82), CD41-FTIC (MWReg30, 11-0411-82), Flt3 (A2F10), CD127-PerCP/Cy5.5 (A7R34, 45-1271-82), Ki-67 PE-Cy7 (clone SolA15, 25-5698-80) were from BioLegend or eBiosciences.

Biotin-c-Mpl/TPOR (AMM2, 10403) was from Immuno-Biological Laboratories (IBL).

Biotinylated lineage cocktail (559971) and FITC anti-active Caspase-3 apoptosis kit (550480) were from BD Biosciences.

pS6 (S235/236; D57.2.2E, 4851S), S6 (54D2, 55594), p4EBP1 (T37/46; 236B4), p44/42 MAPK (T202/Y204; E10, 4375S), pAKT (S473; D9E, 5315S), all from Cell Signalling, were used at 1ug/ml.
All other antibodies were used at 1:100 dilutions unless otherwise indicated.
FlowCellect™ Autophagy LC3 Antibody-based Assay Kit was from EMD Millipore (FCCH100171).

Validation

Anti-MAEA antibodies were validated by flow cytometry using Maeafloxed;Csf1r-Cre conditional knock out mice BM cells. All other commercial antibodies have been validated by the manufacturers and previous studies performed in our studies (ref. 5 and 15).

Animals and other organisms

Policy information about studies involving animals; ARRIVE guidelines recommended for reporting animal research

Laboratory animals

Animals involved in this study are described in Methods section of the manuscript. Briefly, Maeafl/fl mice were generated in P.S.F. laboratory as previously described. Csf1r-iCre mice were a gift from Dr. Jeffrey W. Pollard (University of Edinburgh), and backcrossed onto C57BL/6 background. C57BL/6 (CD45.2) and Bl6-Ly5.1 (CD45.1) mice were purchased from Charles River Laboratories (Frederick Cancer Research Center, Frederick, MD)/NCI or the Jackson Laboratories (B6.SJL-Ptprca Pepcb/BoyJ). Mx1-Cre (B6.Cg-Tg(Mx1-cre) 1Cgn/J) mice were obtained from The Jackson Laboratory and CD169-Cre mice have been previously described. All animals were housed in specific pathogen-free barrier facility under a 12h:12h light/dark cycle, temperature 68-72°F, humidity 40-70% and fed with autoclaved chow. All experimental procedures were approved by the Animal Care and Use Committee of Albert Einstein College of Medicine. All experiments were performed on mice of both genders with littermate controls from the same colony between 6–12 weeks of age unless otherwise indicated.

Wild animals

No wild animals were used in this study

Field-collected samples

No field-collected samples were used in this study

Ethics oversight

We stated in the Methods that all animal procedures were oversight and approved by the Animal Care and Use Committee of Albert Einstein College of Medicine.

Note that full information on the approval of the study protocol must also be provided in the manuscript.

Flow Cytometry

Plots

Confirm that:

- [x] The axis labels state the marker and fluorochrome used (e.g. CD4-FITC).
- [x] The axis scales are clearly visible. Include numbers along axes only for bottom left plot of group (a 'group' is an analysis of identical markers).
- [x] All plots are contour plots with outliers or pseudocolor plots.
- [x] A numerical value for number of cells or percentage (with statistics) is provided.

Methodology

Sample preparation

BM cells were isolated by flushing long bones with 1 mL of ice-cold PEB (phosphate-buffered saline [PBS]/2 mM EDTA/0.5% bovine serum albumin) buffer through a 1-mL syringe (BD) with a 21G needle (BD) into fluorescence-activated cell sorting (FACS) tubes. For intracellular antigen detection, cells were fixed and permeabilized using BD Cytofix/Cytoperm Fixation and Permeabilization kit (#554714) after surface receptor staining and followed by antibody staining for intracellular markers in the Cytoperm/Cytowash buffer. For cell cycle analysis, DNA content was labelled by Hoechst 33342 (Sigma). For phospho-flow, cells were kept on ice upon isolation and immediately fixed in 1.5% PFA for 10 min. Cells were then washed and stained for cell surface markers. After surface staining, the cells were permeabilized with ice-cold acetone for 10 minutes on ice,

washed and stained with the phospho-specific antibodies.

Instrument	Stained sample suspensions were acquired on a LSR II (BD) for analysis and sorted on a BD FACSAria.
Software	Data was acquired using BD FACSDIVA™ Software (V4.1, BD Biosciences) and analyzed using FlowJo.
Cell population abundance	Purity of sorted populations was routinely examined by re-analysis and maintained at >98%.
Gating strategy	For all flow cytometric analysis and sorting, debris and doublets were excluded by forward and side scatters and DAPI (4', 6-diamino-2-phenylindole) staining was used to exclude dead cells. All gating strategies of hematopoietic stem and progenitors were as previously reported.<br>Hematopoietic stem cells (HSCs): lineage (CD3e, B220, Gr-1, CD11b, Ter119) negative, and Ly6A/E(Sca-1)+ CD117 (c-Kit) +CD150+CD48-.<br>Lymphoid-primed multipotent progenitors (LMPPs) are defined as lineage-Sca-1+CD117+ (LSK) Flt3+.<br>Common myeloid progenitors (CMPs) are defined as CD16/32 low CD34+ lineage-Sca-1-CD117+ (LK);<br>Common lymphoid progenitors (CLPs) are defined as lineage-Sca-1low CD117low Flt3+CD127+;<br>Granulocyte-macrophage progenitors (GMPs)are defined as CD16/32 high CD34+ lineage-Sca-1-CD117+ (LK);<br>Megakaryocyte-erythrocyte progenitors (MEP) are defined as CD16/32- CD34- lineage-Sca-1-CD117+ (LK)<br><br>Positive and negative gates were determined by fluorescence minus one or secondary only staining of the same sample for each marker.

☒ Tick this box to confirm that a figure exemplifying the gating strategy is provided in the Supplementary Information.
