# Supplementary material for: MAEA is an E3 ubiquitin ligase promoting autophagy and maintenance of haematopoietic stem cells
Source: Nat Commun. 2021 May 4;12:2522. doi: 10.1038/s41467-021-22749-1 (PMC8097058; doi:10.1038/s41467-021-22749-1)
Supplement: Supplementary file 3 — Source Data [file 41467_2021_22749_MOESM3_ESM.zip › Uncropped gel images.pptx]

## Slide 1
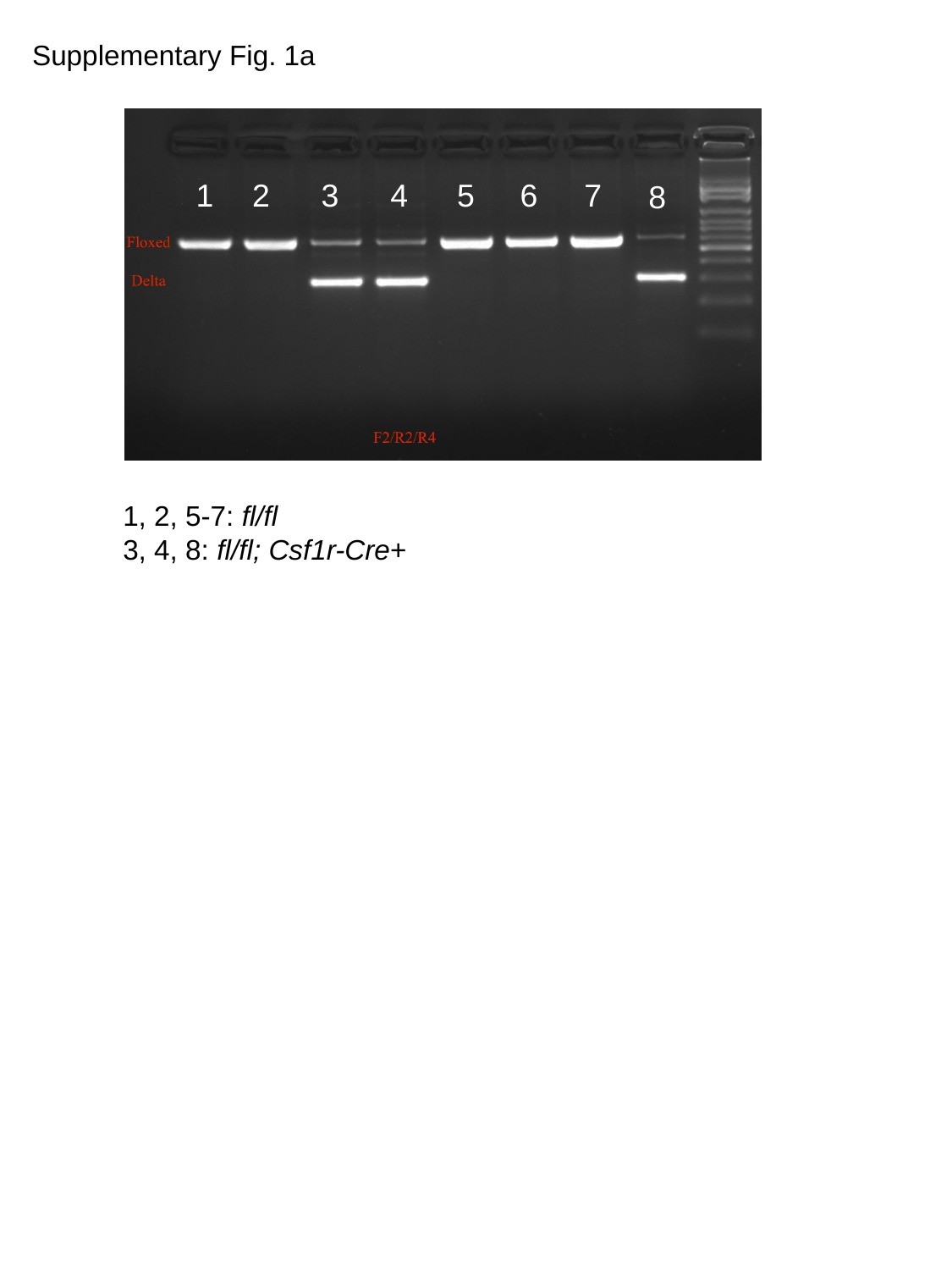

Supplementary Fig. 1a
1
2
4
5
6
7
3
8
1, 2, 5-7: fl/fl
3, 4, 8: fl/fl; Csf1r-Cre+

## Slide 2
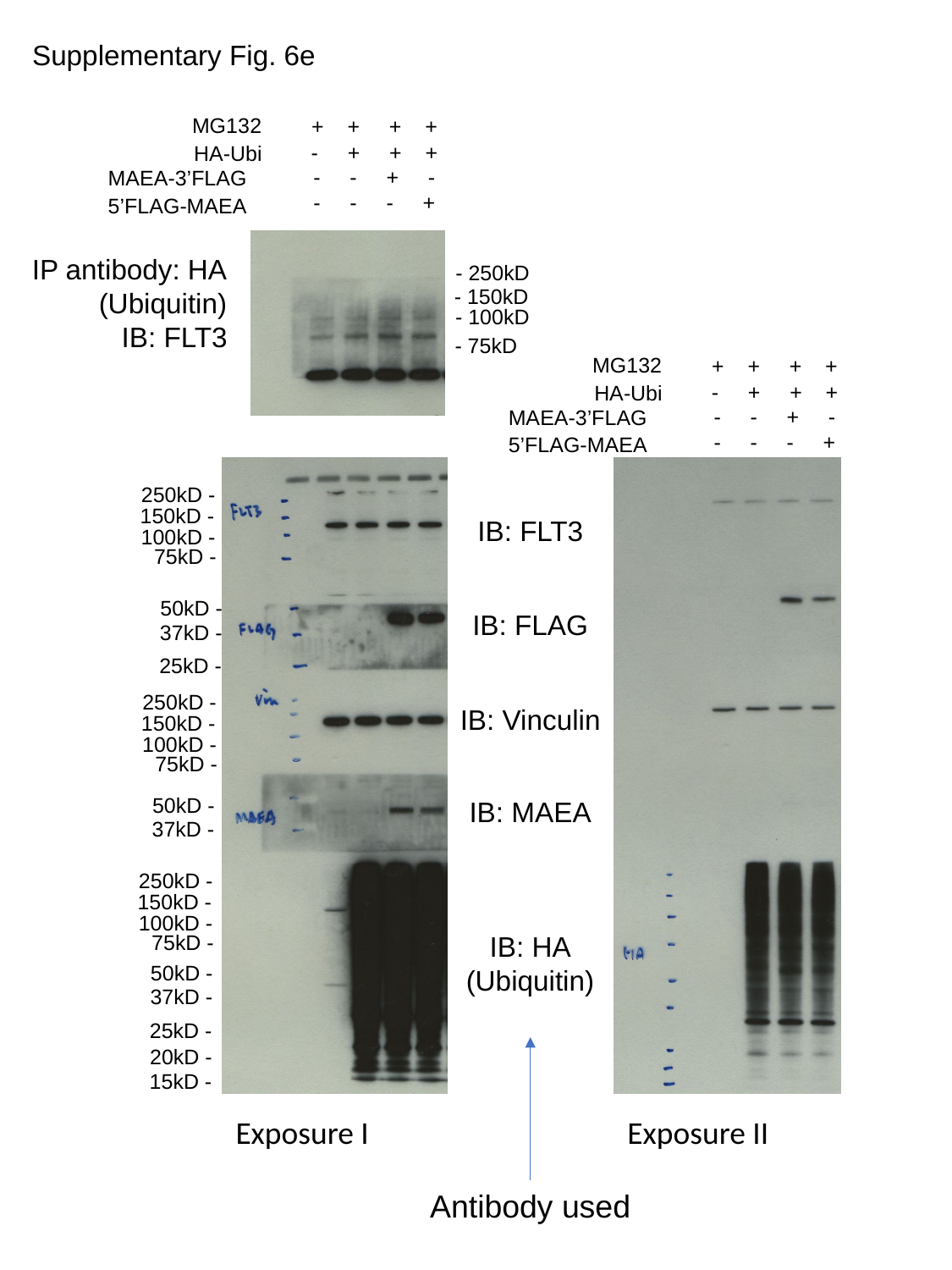

Supplementary Fig. 6e
MG132
+ + + +
- + + +
HA-Ubi
- - + -
MAEA-3’FLAG
- - - +
5’FLAG-MAEA
IP antibody: HA
(Ubiquitin)
IB: FLT3
- 250kD
- 150kD
- 100kD
- 75kD
MG132
+ + + +
- + + +
HA-Ubi
- - + -
MAEA-3’FLAG
- - - +
5’FLAG-MAEA
250kD -
150kD -
100kD -
75kD -
50kD -
37kD -
25kD -
250kD -
150kD -
100kD -
75kD -
50kD -
37kD -
250kD -
150kD -
100kD -
75kD -
50kD -
37kD -
25kD -
20kD -
15kD -
IB: FLT3
IB: FLAG
IB: Vinculin
IB: MAEA
IB: HA
(Ubiquitin)
Exposure II
Exposure I
Antibody used
